# Influence of Age on the Diagnosis of Myocardial Infarction

**DOI:** 10.1161/CIRCULATIONAHA.122.059994

**Published:** 2022-09-15

**Authors:** Matthew T.H. Lowry, Dimitrios Doudesis, Ryan Wereski, Dorien M. Kimenai, Christopher Tuck, Amy V. Ferry, Anda Bularga, Caelan Taggart, Kuan K. Lee, Andrew R. Chapman, Anoop S.V. Shah, David E. Newby, Nicholas L. Mills, Atul Anand

**Affiliations:** BHF Centre for Cardiovascular Science (M.T.H.L., D.D., R.W., D.M.K., C.T., A.V.F., A.B., C.T., K.K.L., A.R.C., D.E.N., N.L.M., A.A.), University of Edinburgh, UK.; Usher Institute (D.D., N.L.M.), University of Edinburgh, UK.; Department of Non-communicable Disease, London School of Hygiene and Tropical Medicine, UK (A.S.V.S.).; Department of Cardiology, Imperial College Healthcare NHS Trust, London, UK (A.S.V.S.).

**Keywords:** acute coronary syndrome, aging, frail elderly, myocardial infarction, troponin

## Abstract

The 99th centile of cardiac troponin, derived from a healthy reference population, is recommended as the diagnostic threshold for myocardial infarction, but troponin concentrations are strongly influenced by age. Our aim was to assess the diagnostic performance of cardiac troponin in older patients presenting with suspected myocardial infarction.

**Methods:**

In a secondary analysis of a multicenter trial of consecutive patients with suspected myocardial infarction, we assessed the diagnostic accuracy of high-sensitivity cardiac troponin I at presentation for the diagnosis of type 1, type 2, or type 4b myocardial infarction across 3 age groups (<50, 50–74, and ≥75 years) using guideline-recommended sex-specific and age-adjusted 99th centile thresholds.

**Results:**

In 46 435 consecutive patients aged 18 to 108 years (mean, 61±17 years), 5216 (11%) had a diagnosis of myocardial infarction. In patients <50 (n=12 379), 50 to 74 (n=22 380), and ≥75 (n=11 676) years, the sensitivity of the guideline-recommended threshold was similar at 79.2% (95% CI, 75.5–82.9), 80.6% (95% CI, 79.2–82.1), and 81.6% (95% CI, 79.8–83.2), respectively. The specificity decreased with advancing age from 98.3% (95% CI, 98.1–98.5) to 95.5% (95% CI, 95.2–95.8), and 82.6% (95% CI, 81.9–83.4). The use of age-adjusted 99th centile thresholds improved the specificity (91.3% [90.8%–91.9%] versus 82.6% [95% CI, 81.9%–83.4%]) and positive predictive value (59.3% [57.0%–61.5%] versus 51.5% [49.9%–53.3%]) for myocardial infarction in patients ≥75 years but failed to prevent the decrease in either parameter with increasing age and resulted in a marked reduction in sensitivity compared with the use of the guideline-recommended threshold (55.9% [53.6%–57.9%] versus 81.6% [79.8%–83.3%].

**Conclusions:**

Age alters the diagnostic performance of cardiac troponin, with reduced specificity and positive predictive value in older patients when applying the guideline-recommended or age-adjusted 99th centiles. Individualized diagnostic approaches rather than the adjustment of binary thresholds are needed in an aging population.

**Clinical Perspective**

**What Is New?**

- In older patients presenting with suspected myocardial infarction, the majority of cardiac troponin elevations are explained by acute or chronic myocardial injury or type 2 myocardial infarction.
- The specificity and positive predictive value of high-sensitivity cardiac troponin to identify myocardial infarction decreases with age and is observed whether applying sex-specific or age-adjusted 99th centile diagnostic thresholds or a “rule-in” threshold for the triage of patients at high probability of myocardial infarction.
- Serial troponin testing incorporating an absolute change in troponin concentration increased discrimination for myocardial infarction in older patients.

**What Are the Clinical Implications?**

- In older patients presenting with suspected myocardial infarction, clinicians should be cautious when interpreting a single troponin measurement.
- Clinicians should routinely perform serial cardiac troponin measurements and consider absolute changes in concentration to identify those older patients with elevated troponin concentrations more likely to have myocardial infarction.

The 99th centile upper reference limit (URL) of cardiac troponin, derived from a cohort of healthy individuals, is used as the threshold to indicate myocardial injury and potential infarction.^1^ This value is influenced by the characteristics of the reference population used for derivation.^2–5^ Elevated concentrations of cardiac troponin >99th centile are frequently observed in older adults,^3,4,6–8^ including among those presenting to the emergency department without myocardial infarction^9–11^ and in the general hospitalized older population.^12^ The application of diagnostic thresholds derived from younger reference populations may incorrectly suggest myocardial infarction in older patients, resulting in inappropriate treatment and potential harm.

The relationship between age and cardiac troponin has been noted for both troponin I and T assays, with the observed 99th centile URL for older adults in the general population double the reference value for cardiac troponin I and 3 times the value for troponin T.^3^ Cardiovascular comorbidities including hypertension, diabetes, left ventricular dysfunction, and existing ischemic heart disease are independently associated with chronic elevations in cardiac troponin.^3,4,6,7,9^ The higher prevalence of these conditions among older patients further complicates the interpretation of cardiac troponin in an aging and increasingly multimorbid society.

Age-adjusted thresholds that use the observed 99th centile within different age groups to guide the diagnosis have been proposed as a means of increasing the specificity of cardiac troponin for myocardial infarction in older patients.^13–15^ An alternative strategy to increase the specificity is the use of a threshold >99th centile. Introduced in recent practice guidelines, direct rule-in approaches using the presentation troponin concentration and a threshold ≈3 times the 99th centile value to identify patients at high probability of myocardial infarction are reported to have greater specificity and a positive predictive value (PPV) of up to 75%.^14^

Previous evaluations on the effect of age when applying either strategy have focused on the identification of any form of myocardial infarction.^8,11,16,17^ Although both type 1 and type 2 myocardial infarctions represent important clinical entities, they have divergent treatment strategies, and an understanding of how age affects diagnostic performance specifically for type 1 myocardial infarction would help guide treatment decisions in older patients.

In this prespecified secondary analysis of a multicenter trial of consecutive patients with suspected acute coronary syndrome, we evaluate the effect of age and cardiovascular comorbidities on the performance of high-sensitivity cardiac troponin I for the diagnosis of myocardial infarction using the guideline-recommended sex-specific 99th centile, age-adjusted sex-specific 99th centiles derived in a general population, and a universal rule-in threshold >99th centile. In addition, we assess the performance of each threshold in combination with absolute and relative change in troponin concentration for the diagnosis of myocardial infarction.

## Methods

### Study Population

The High-STEACS trial (High-Sensitivity Troponin in the Evaluation of Patients with Suspected Acute Coronary Syndrome) is a stepped-wedge cluster randomized controlled trial that evaluated the implementation of a high-sensitivity cardiac troponin I assay in consecutive patients presenting with suspected acute coronary syndrome across 10 secondary and tertiary hospitals in Scotland (https://www.clinicaltrials.gov; Unique identifier: NCT01852123). A detailed description of this trial has been reported previously.^18^ In summary, all patients attending the emergency department between June 2013 and March 2016 in whom the attending clinician suspected acute coronary syndrome and underwent cardiac troponin sampling were considered eligible for inclusion. Patients were excluded if they had been admitted previously during the trial period or did not reside in Scotland. Patients were enrolled using an electronic form integrated into the clinical care pathway completed at the time of cardiac troponin sampling.

For this secondary analysis, patients with ST-segment–elevation myocardial infarction, those in whom the presentation high-sensitivity cardiac troponin sample was unavailable, those with an adjudicated diagnosis of type 4a myocardial infarction, or in whom a final diagnosis could not be adjudicated, were excluded.

### Cardiac Troponin Testing

Cardiac troponin testing was performed at presentation and repeated 6 or 12 hours after the onset of symptoms at the discretion of the attending clinician in accordance with international guidelines in use during enrollment.^19^ Cardiac troponin was measured using the ARCHITECT_STAT_ high-sensitivity troponin I assay (Abbott Laboratories). This assay has a limit of detection of between 1.2 and 1.9 ng/L, an interassay coefficient of variation of <10% at 4.7 ng/L, and a 99th centile URL of 34 ng/L in men and 16 ng/L in women. Sex-specific URL was determined by the manufacturer on the basis of 4590 samples from healthy men and women aged 21 to 75 years.^20^

### Diagnostic Adjudication

All patients with a high-sensitivity cardiac troponin I concentration >99th centile were adjudicated and classified according to the Fourth Universal Definition of Myocardial Infarction.^1,18,21^ Two physicians independently reviewed all clinical information, with discordant diagnoses resolved by an independent third physician. Type 1 myocardial infarction was defined as myocardial necrosis (any high-sensitivity cardiac troponin I concentration above the sex-specific 99th percentile with a rise or fall in troponin where serial testing was performed) in the context of a presentation with suspected acute coronary syndrome and symptoms or signs of myocardial ischemia. Patients with myocardial necrosis, symptoms or signs of myocardial ischemia, and evidence of increased myocardial oxygen demand or decreased supply secondary to an alternative condition without evidence of acute atherothrombosis were defined as type 2 myocardial infarction. Type 4a myocardial infarction was defined in patients with symptoms or signs of myocardial ischemia after percutaneous coronary intervention where high-sensitivity cardiac troponin I concentrations were 5-fold greater than the 99th centile or increased further if elevated before the procedure. Type 4b myocardial infarction was defined where myocardial ischemia and myocardial necrosis were associated with stent thrombosis documented at angiography. Patients with high-sensitivity cardiac troponin I concentrations above the 99th centile without symptoms or signs of myocardial ischemia were classified as having myocardial injury. All nonischemic myocardial injury was classified as acute, unless a change of ≤20% was observed on serial testing,^1^ or the final adjudicated diagnosis was chronic heart failure or chronic renal failure, where the classification was chronic myocardial injury. The term “myocardial infarction” is used to denote patients with an adjudicated diagnosis of type 1, type 2, or type 4b myocardial infarction. A detailed summary of the adjudication process is provided in the Supplemental Material.

### Statistical Analysis

Baseline characteristics are summarized as number (%) for categorical variables, and continuous variables are summarized as mean±SD or median (25th to 75th percentile) when not normally distributed. The study population was divided into 3 clinically relevant age groups: young (<50 years), middle-aged (50–74 years), and older adults (≥75 years). For additional analyses, the population was divided by 5-year intervals between the ages of 40 and 90 years to create 12 groups. Patients aged <40 and ≥90 years were pooled into groups of <40 and ≥90 years, respectively. Groupwise comparisons were performed using χ^2^, Kruskal-Wallis, or 1-way ANOVA tests as appropriate.

We evaluated the proportion of patients with at least one troponin concentration above the sex-specific 99th centile URL for each age category. Diagnostic performance was assessed by using sensitivity, specificity, negative predictive value, and PPV, and calculated using a 2×2 confusion matrix. Corresponding 95% CIs were calculated using bootstrapping with replacement and a sample of 1000. We calculated diagnostic performance for a high-sensitivity cardiac troponin I concentration at presentation above the guideline-recommended sex-specific 99th centile (16 ng/L women, 34 ng/L men),^1^ age-adjusted 99th centile thresholds in patients >60 years (age <60 years=34 ng/L men, 16 ng/L women; age 60–69 years=42 ng/L men, 17 ng/L women; age ≥70 years=86 ng/L men, 39 ng/L women), and a universal rule-in threshold (64 ng/L) recommended by the European Society of Cardiology.^14^ Age-adjusted thresholds were previously derived in 19 501 individuals in the Generation Scotland Scottish Family Health Study.^3^ Overall performance was assessed using area under the curve (AUC) and compared between thresholds and age groups using a DeLong test.

A sensitivity analysis was undertaken using the 99th centile as the diagnostic threshold restricted to patients presenting with chest pain. Additional analysis restricted to patients with serial samples taken within 24 hours of admission was performed to assess the effect of the change in cardiac troponin concentration from serial samples on diagnostic performance. We evaluated models that incorporated absolute or relative change in troponin concentration of 15 ng/L or 20% as recommended in international guidelines in combination with the presentation troponin concentration stratified by age group and threshold.^14,15,22^ The effect of change in cardiac troponin concentration on discrimination was assessed using the AUC and compared between thresholds and age groups using a DeLong test.^1^

Logistic regression was used to explore the influence of cardiovascular comorbidities on the probability of myocardial infarction given a cardiac troponin value greater than the sex-specific 99th centile. A history of ischemic heart disease, myocardial infarction, heart failure, cerebrovascular disease (defined as previous ischemic or hemorrhagic stroke), chronic renal impairment (defined as an estimated glomerular filtration rate <60 mL·min^–1^·1.73 m^–2^ determined by the Modified Diet in Renal Disease equation) and diabetes were added individually (model 1) and collectively (model 2) to a baseline model, including a binary explanatory variable of presentation troponin greater than the sex-specific 99th centile. Collinearity was assessed visually and by calculation of the generalized variance inflation factor. All analyses were performed in R Version 3.5.1.

### Ethical Approval

The study was approved by the Scotland Research Ethics Committee, the Public Benefit and Privacy Panel for Health and Social Care, and each National Health Service Health Board. Individual patient consent was not required, and data from consecutive patients were collected prospectively from the electronic record, deidentified, and linked within secure National Health Service Safe Havens.

### Patient and Public Involvement

Patients and lay representatives were members of the steering committee for the trial and all related studies and were involved in the design, conduct, and approval of this study.

## Results

A total of 46 435 of the 48 282 patients enrolled in the trial were included in the analysis population. Patients with ST-segment–elevation myocardial infarction (n=925), those in whom the final diagnosis could not be adjudicated according to the Fourth Universal Definition of Myocardial Infarction (n=890), those with an adjudicated diagnosis of type 4a myocardial infarction (n=9), and those without a presentation high-sensitivity cardiac troponin result (n=23) were excluded.

### Baseline Characteristics

Participants were aged between 18 and 108 years (mean, 61±17 years). Baseline characteristics for the population are shown in Table 1 (Table S1). Compared with younger patients, those aged ≥75 years were more often female and less likely to present with chest pain or ischemia on 12-lead ECG (*P*<0.001 for all). There was a higher prevalence of cardiovascular comorbidity in patients ≥75 years, including ischemic heart disease, heart failure, diabetes, and chronic kidney disease (*P*<0.001 for all). More than half of patients ≥75 years had ≥2 chronic cardiovascular health conditions compared with a third aged between 50 and 74 years (56% versus 32%, respectively, *P*<0.001).

**Table 1.**
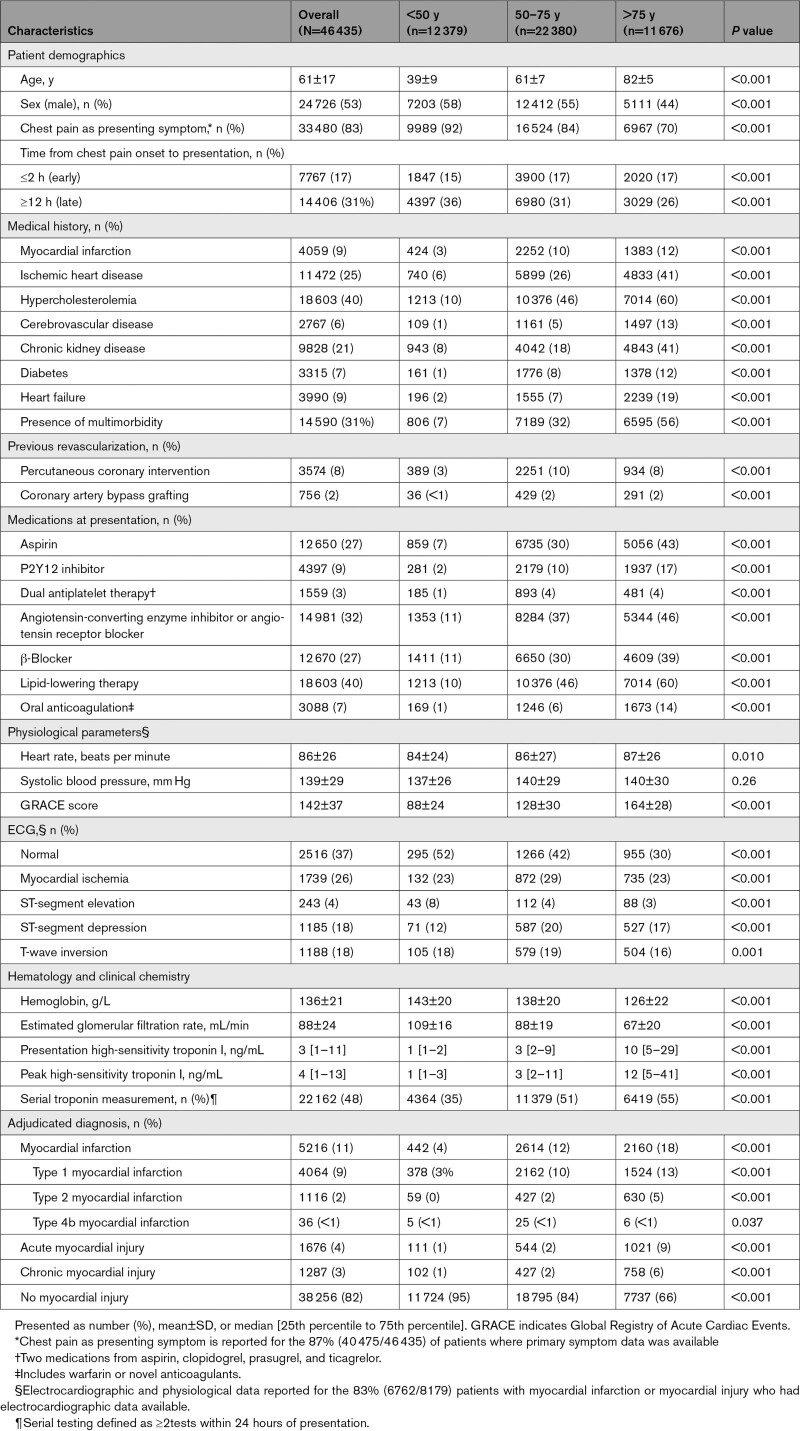
Baseline Characteristics Stratified by Age Group

**Figure 1.**
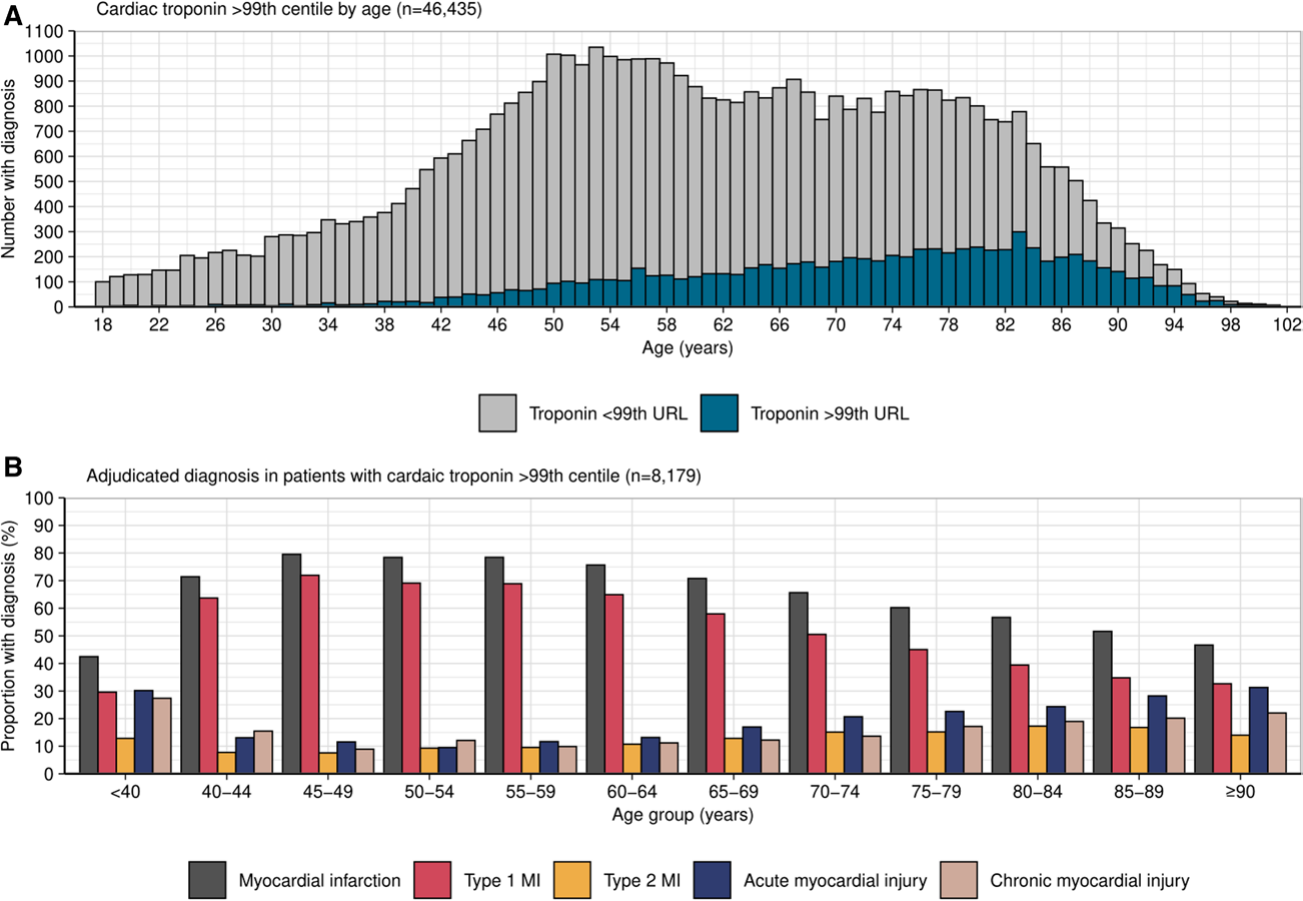
**Cardiac troponin testing and adjudicated diagnosis by age. A**, Histogram showing the number of patients with 1 cardiac troponin concentration above the sex-specific 99th centile by age in all study patients. The number of patients with a cardiac troponin above the sex-specific 99th centile increases with age (n=46 435). **B**, Bar chart showing adjudicated diagnoses in patients with 1 cardiac troponin value above the 99th centile as a proportion of each age group. With advancing age, the proportion with type 1 myocardial infarction decreases as non–type 1 infarction and myocardial injury increase (n=8179). MI indicates myocardial infarction; and URL, upper reference limit.

A total of 8179 (18%) patients had at least one cardiac troponin measurement above the sex-specific 99th centile. For those aged <50, 50 to 74, and ≥75 years, the proportion of patients with at least one measure above the sex-specific 99th centile was 5%, 16%, and 34%, respectively (*P*<0.001 for difference between groups). In patients aged ≥90 years, 49% had one cardiac troponin above the sex-specific 99th centile (Figure S1). Myocardial infarction was the final adjudicated diagnosis in 5216 (11%) patients with the prevalence highest in those aged ≥75 years (18%). In patients with at least one troponin measurement greater than the sex-specific 99th centile, the proportion of those with type 1 myocardial infarction decreased with advancing age as type 2 myocardial infarction, acute myocardial injury, and chronic myocardial injury increased (Figure 1).

### Diagnostic Performance of the 99th Centile at Presentation

**Figure 2.**
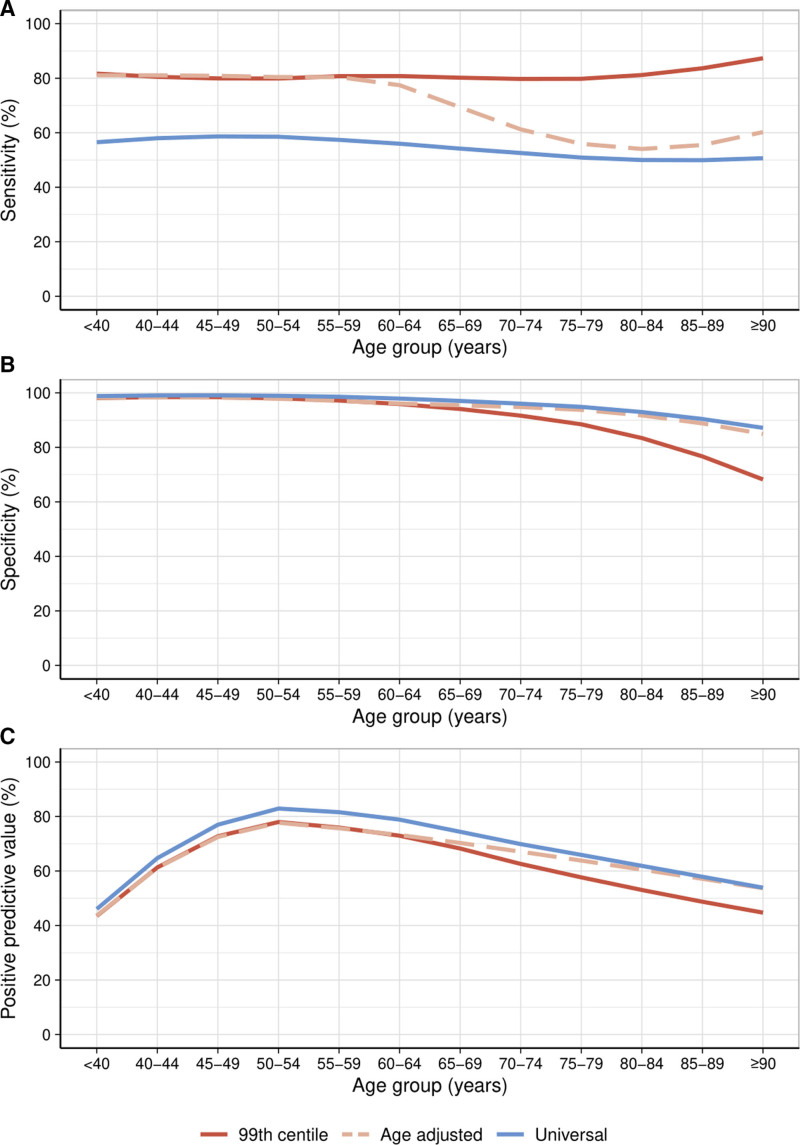
**Diagnostic performance of the sex-specific 99th centile and alternative thresholds.** The sensitivity (**A**), specificity (**B**), and positive predictive value (**C**) of the recommended sex-specific 99th centile, age-adjusted thresholds, and a universal rule-in threshold above the 99th centile across age groups plotted with a line of best fit.

In patients aged <50, 50 to 74, and ≥75 years, the sensitivity of the guideline-recommended sex-specific 99th centile at presentation for a diagnosis of myocardial infarction was similar at 79.2% (95% CI, 75.5–82.9), 80.6% (95% CI, 79.2–82.1), and 81.6% (95% CI, 79.8–83.2), respectively. The specificity fell with advancing age from 98.3% (95% CI, 98.1–98.5) to 95.5% (95% CI, 95.2–95.8), and 82.6% (95% CI, 81.9–83.4) for those aged <50, 50 to 74, and ≥75 years, respectively. The PPV for those aged <50, 50 to 74, and ≥75 years was 63.0% (95% CI, 59.1–67.1), 70.1% (95% CI, 68.5–71.8), and 51.6% (95% CI, 49.8–53.2), respectively (Table 2, Figure 2, Table S2).

**Table 2.**
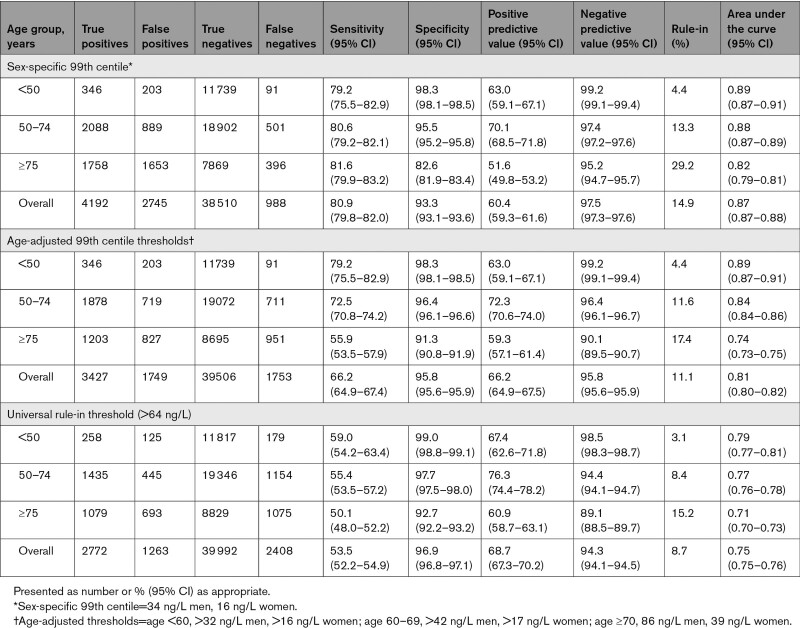
Diagnostic Performance of Presentation High-Sensitivity Cardiac Troponin I for Myocardial Infarction by Age Group and Threshold

In a sensitivity analysis restricted to those with chest pain at presentation (n=33 446), the sensitivity for myocardial infarction was similar to patients presenting with any symptom, whereas specificity and PPV were markedly increased across all age groups. In patients ≥75 years, the specificity and PPV were 89.8% (95% CI, 89.0–90.6) and 70.4% (95% CI, 68.5–72.4), respectively (Figure S2, Table S3).

### Diagnostic Performance of Age-Adjusted 99th Centile Thresholds

Applying age-adjusted thresholds resulted in higher specificity and PPV for myocardial infarction in patients ≥75 years at the expense of a marked reduction in sensitivity (Table 2, Figure 2, Table S2). In patients ≥75 years, sensitivity, specificity, and PPV were 55.9% (95% CI, 53.5–57.9), 91.3% (95% CI, 90.8–91.9), and 59.3% (95% CI, 57.1–61.4), respectively. Despite the use of age-adjusted thresholds, the specificity and PPV remained lower in patients ≥75 years than in patients <50 or 50 to 74 years old. Compared with the guideline-recommended sex-specific 99th centile, discrimination was reduced (AUC, 0.81 [95% CI, 0.80–0.82] versus 0.87 [95% CI, 0.87–0.88], *P*<0.001).

### Diagnostic Performance of a Universal Rule-in Threshold Above the 99th Centile

Applying a universal rule-in threshold of 64 ng/L resulted in increased specificity and PPV for myocardial infarction, with reduced sensitivity across all age groups, compared with sex-specific or age-adjusted 99th centile thresholds (Table 2, Figure 2, Table S2). In patients ≥75 years, sensitivity, specificity, and PPV were 50.1% (95% CI, 48.0–52.2), 92.7% (95 % CI, 92.2–93.2), and 60.9% (95% CI, 58.7–63.1), respectively. Specificity and PPV remained lower in patients ≥75 years than in those <50 or 50 to 74 years. Compared with the guideline-recommended sex-specific 99th centile, discrimination was reduced (AUC 0.75 [95% CI, 0.75–0.76] versus 0.87 [95% CI, 0.87–0.88], *P*<0.001).

### Diagnostic Performance of Serial Measurements

In a sensitivity analysis restricted to those with serial samples taken within 24 hours of admission {n=20 881 (age <50, 3962 [19%]; age 50–74, 10 826 [52%]; age ≥75, 6093 [29%])} both a relative change of 20% and absolute change of 15 ng/L significantly improved discrimination across all groups compared with a presentation sample alone (*P*<0.001 for all; Table 3). In patients aged ≥75 years, an age-adjusted threshold in combination with an absolute delta of 15 ng/L achieved the greatest discrimination (AUC, 0.94 [95% CI, 0.93–0.95]) compared with the sex-specific 99th centile or universal rule-in threshold (0.88 [95% CI, 0.87–0.89] and 0.82 [95% CI, 0.81–0.83], respectively). Overall, discrimination was greatest when applying the sex-specific 99th centile with an absolute change of 15 ng/L compared with the application of this delta criterion in combination with either an age-adjusted or universal rule-in threshold (*P*<0.001 for both).

**Table 3.**
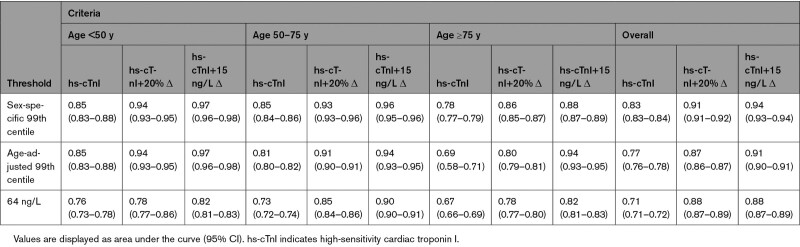
Discrimination of High-Sensitivity Cardiac Troponin I at Presentation in Combination With an Absolute or Relative Change in Cardiac Troponin Concentration

### Effect of Cardiovascular Comorbidity on Diagnostic Performance

An elevated troponin above the 99th centile was associated with myocardial infarction across all age groups, but this relationship was weakest in patients ≥75 years (Table 4). Several cardiovascular comorbidities were strongly associated with myocardial infarction and altered the PPV of a presentation troponin above the sex-specific 99th centile for myocardial infarction (Figure S3). Adjusting for cardiovascular comorbidities did not alter the association between a high-sensitivity cardiac troponin above the 99th centile and a diagnosis of myocardial infarction but did improve overall discrimination across all age groups (age <50 years [*P*=0.003]; age 50–74 years [*P*<0.001]; age ≥75 years [*P*<0.001]).

**Table 4.**
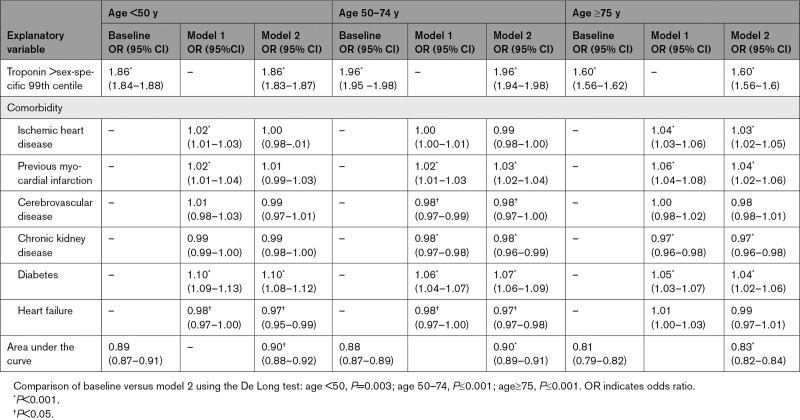
Logistic Regression Models for Determinants of Myocardial Infarction

### Sensitivity Analysis of Diagnostic Performance for Type 1 Myocardial Infarction

**Figure 3.**
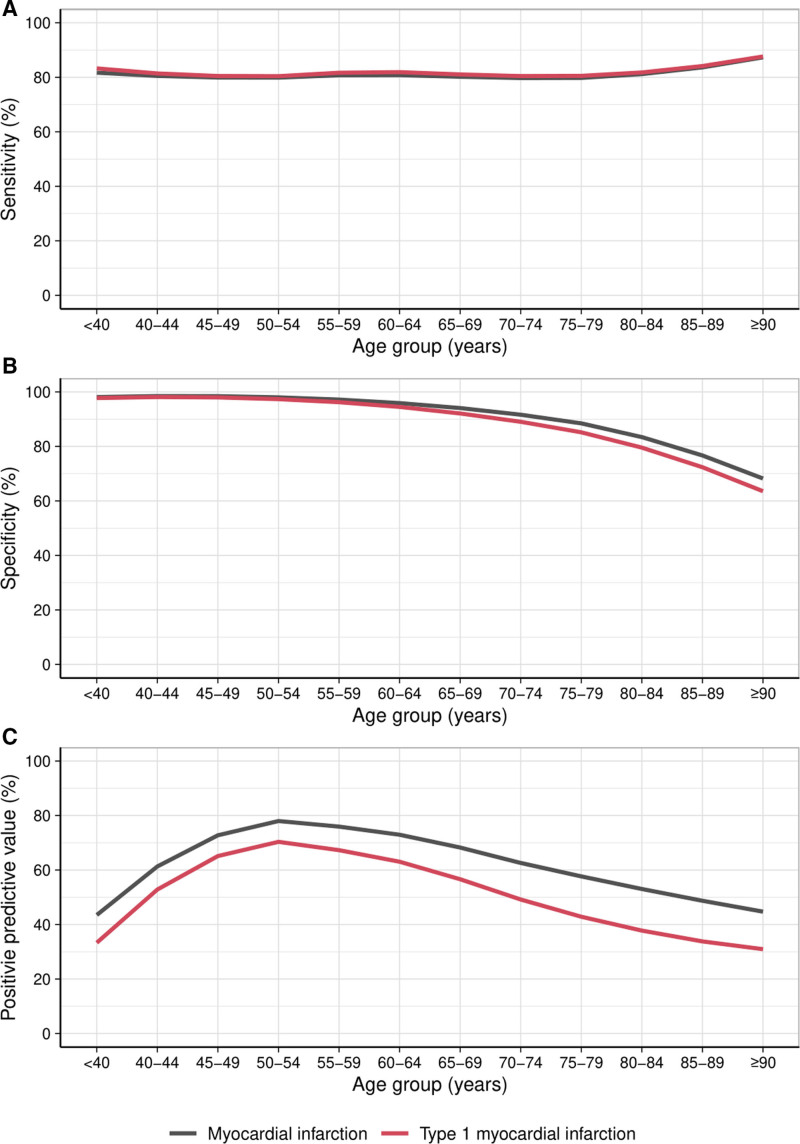
**Diagnostic performance of the sex-specific 99th centile for the diagnosis of type 1 myocardial infarction.** The sensitivity (**A**), specificity (**B**), and positive predictive value (**C**) of the recommended sex-specific 99th centile for the diagnosis of type 1 myocardial infarction (red) compared with any myocardial infarction (black) plotted with a line of best fit.

Compared with a diagnosis of any type of myocardial infarction, assessing the diagnostic performance of each threshold specifically for type 1 myocardial infarction resulted in similar sensitivity across all age groups with reduced specificity and PPV, in particular, in older patients. Using the guideline-recommended sex-specific 99th centile, specificity and PPV in patients ≥75 years was 78.8% (95% CI, 78.0–79.6) and 36.8% (95% CI, 35.0–38.3), respectively (Figure 3, Table S4).

## Discussion

We report the effect of our aging population on the diagnostic challenge facing clinicians evaluating patients with suspected acute coronary syndrome. Our analysis is informed by 46 435 consecutive patients, aged 18 to 108, and we report several important findings. First, cardiac troponin concentrations above the recommended sex-specific 99th centile are common in older patients, affecting almost half of those aged >90 years. In older age groups, the majority of cardiac troponin elevations are explained by acute or chronic myocardial injury or type 2 myocardial infarction. Second, the specificity and PPV of the guideline-recommended 99th centile for diagnosing myocardial infarction decreases with advancing age. The decrease in these parameters is more pronounced when restricting the diagnosis to type 1 myocardial infarction. Third, the use of an age-adjusted 99th centile or a universal rule-in threshold of 64 ng/L resulted in superior specificity and PPV for myocardial infarction compared with the sex-specific 99th centile, with a threshold of 64 ng/L achieving the greatest improvement in these parameters. However, no approach achieved parity in the diagnosis between older and younger patients with specificity and PPV reducing with advancing age regardless of the threshold adopted, and alternatives to the guideline-recommended approach resulted in a marked reduction in sensitivity in older persons. Fourth, although cardiovascular comorbidities are common in older patients and related to a diagnosis of myocardial infarction, they did not alter the strength of association between an elevated cardiac troponin and the diagnosis. Fifth, serial troponin testing incorporating an absolute change in troponin concentration increased discrimination for myocardial infarction in older patients and was superior to any single test strategy. Our findings highlight the challenge of interpreting elevated cardiac troponin concentrations in older adults and the limitations of single test strategies to rule-in myocardial infarction in this population.

The majority of patients diagnosed with myocardial infarction are aged >70 years.^23^ With an aging population, these numbers will continue to rise. Our observation of complexity among older patients, notably the higher frequency of atypical symptoms and nondiagnostic ECG findings, may result in clinicians placing greater reliance on the potential objectivity of blood biomarkers of myocardial necrosis. We observed a decrease in chest pain as a presenting symptom in older patients and have previously reported that many older patients with myocardial infarction do not present with chest pain.^18^ We included all patients in whom a clinician suspected acute coronary syndrome, including 6995 (17%) patients in whom the primary presenting symptom was not chest pain. For meaningful interpretation of the diagnostic performance of cardiac troponin, it is important that assessments are performed in study populations representative of those seen in clinical practice. Selective inclusion criteria that result in the exclusion of older patients reduce generalizability and risks mirroring previous biases that resulted in the systematic underdiagnosis of myocardial infarction in women.^20^

Our finding of reduced specificity of the sex-specific 99th centile in older patients is consistent with previous literature assessing both sensitive and high-sensitivity assays for the diagnosis of myocardial infarction.^8,11,24^ Reiter et al^11^ compared the performance of sensitive troponin assays between patients >70 and <70 years using a cohort of 1098 patients from the APACE study (Advantageous Predictors of Acute Coronary Syndromes Evaluation). Boeddinghaus et al^16^ assessed the effect of age on the performance a 0/1 hour chest pain pathway using the 99th centile diagnostic threshold for both high-sensitivity cardiac troponin I and T assays in a cohort of 3123 patients from APACE, BACC (Biomarkers in Acute Cardiac Care), and TRAPID-MI (High Sensitivity Cardiac Troponin T assay for rapid Rule-out of Acute Myocardial Infarction) with chest pain. Both studies reported that specificity for myocardial infarction decreased with advancing age.

We found that the use of age-adjusted thresholds improved specificity and PPV in older patients than the use of the 99th centile, a finding mirrored in several observational studies.^8,11,16^ Reclassification of patients using an age-adjusted diagnostic threshold has also been shown to improve the identification of patients at increased short-term mortality.^17^ Parallels could be drawn with the use of sex-specific thresholds that are recommended in the Fourth Universal Definition of Myocardial Infarction.^1,20^ Is it therefore time to consider adopting age-adjusted thresholds? There are several factors to consider. First, age is not a dichotomous variable. Deriving the 99th centile in a population by age still confers the same issues inherent with a universal 99th centile: defining normality in a heterogeneous group. Second, higher cardiac troponin thresholds may disadvantage older patients with fewer comorbidities. Third, elevated cardiac troponin levels above the 99th centile are associated with adverse outcomes in both young and old patients and implementing higher thresholds may normalize values that still confer risk, limiting the opportunity for intervention.^25^ Fourth, age-adjusted 99th centiles did not prevent a decline in diagnostic performance of troponin testing in older patients. Last, overall discrimination was greatest when using an absolute change in cardiac troponin in combination with the 99th centile as the diagnostic threshold. For these reasons, we do not support the adoption of age-adjusted thresholds for the diagnosis of myocardial infarction.

The latest European Society of Cardiology guidelines have included new rule-in thresholds above the 99th centile to identify those with a high probability of myocardial infarction using a single presentation cardiac troponin test.^14^ This extends the concept of safety from a single low cardiac troponin concentration to an idea that high presentation concentrations are very likely to correlate with the severity of coronary artery disease.^25,26^ Rule-in thresholds were designed to maximize the specificity and PPV of testing with their recommendation based on observational data from cohorts of consented patients with chest pain as the primary presenting symptom.^16,24,28^ We found that the application of a rule-in threshold of 64 ng/L achieved the greatest specificity and PPV for both myocardial infarction and type 1 myocardial infarction across all ages in comparison with both sex-specific 99th centiles and age-adjusted thresholds. This approach is analogous to the use of optimized rule-out or risk stratification thresholds that prioritize high sensitivity and negative predictive value to identify patients at presentation who are unlikely to have myocardial infarction on serial testing. However, unlike these thresholds, we observed that a rule-in threshold did not have consistent or adequate performance across age groups or key cardiovascular comorbidities. Despite higher specificity and PPV, 2 in every 5 patients aged ≥75 years with a presentation cardiac troponin >64 ng/L did not have myocardial infarction, and 1 in every 2 patients aged 75 years did not have a final diagnosis of type 1 myocardial infarction. In addition, sensitivity was decreased across all age groups. This may miss diagnoses of myocardial infarction and other forms of myocardial injury that confer clinically relevant and prognostic information.^29^ Ultimately, any increase in a binary threshold comes at the cost of decreased sensitivity, regardless of age. Although defining optimal thresholds for a series of age groups and comorbidities to achieve a predefined specificity or PPV may be possible, these would be impractical to apply in clinical practice.

Regardless of threshold, diagnostic performance was reduced in older patients. We observed an increase in type 2 myocardial infarction and myocardial injury with age. Cardiac troponin is not specific for myocardial infarction, and there is little evidence that the magnitude of cardiac troponin can distinguish the mechanism of release, and the differentiation of acute from chronic causes of injury requires serial testing.^1,30–34^ Given the ease of access to early retesting within 1 hour, and the improvements in diagnostic performance when incorporating an absolute change in troponin concentration, clinicians should consider whether the rule-in of myocardial infarction on the basis of a single presentation cardiac troponin sample should be applied to older or more complex patients. Patients requiring immediate or expedited revascularization are often identifiable by clinical features, and decisions based on presentation troponin concentrations should first focus on safe rule-out and minimizing the risk of missed myocardial infarction.

We observed a lower specificity and PPV when using high-sensitivity cardiac troponin to diagnose type 1 myocardial infarction compared with a diagnosis of type 1, type 2, or type 4b infarction. Although chest pain diagnostic pathways predominately assist with patient triage, they are also used to guide the early administration of antiplatelet therapy and anticoagulation that are not indicated in patients with type 2 myocardial infarction and conversely may cause harm. Clinicians should be aware of these changes when considering the risks and benefits of early management strategies in older patients.

Few studies have assessed the effect of comorbidities on diagnostic performance of troponin testing. We found that, although several cardiovascular comorbidities were associated with the diagnosis of myocardial infarction, their presence did not alter the odds of myocardial infarction in those with an elevated cardiac troponin above the 99th centile. This suggests that the cardiovascular comorbidities we assessed do not directly influence the diagnostic performance of a binary rule-in strategy using cardiac troponin at the sex-specific 99th centile. There are several potential explanations for these findings. First, older patients free from cardiovascular disease may still exhibit higher baseline cardiac troponin concentrations than younger reference populations used to derive 99th centile thresholds.^5,6^ Age may therefore have a stronger association with cardiac troponin concentrations than individual comorbidities. Second, noncardiovascular comorbidities were not collected as part of the High-STEACS trial. Conditions such as chronic obstructive pulmonary disease and other inflammatory conditions are associated with elevations in cardiac troponin.^9,35–37^ Third, we cannot exclude the effect of unmeasured subclinical cardiovascular disease in our cohort. Objective measures of disease severity such as natriuretic peptide concentrations or echocardiography could add to the granularity of a binary comorbidity status. Approaches to sequentially exclude patients from reference populations used to derive the 99th centile using such testing has been shown to affect the threshold level, in particular, in older patients.^8,38,39^

Of note, the addition of comorbidities to our baseline model resulted in an improvement in model discrimination, suggesting that approaches which consider multiple individual patient factors could offer an alternative to threshold-based diagnosis.^40^ One such example is the myocardial infarction^3^ model that uses machine learning to provide individual probability estimates and has been shown to perform favorably in an observational study with superior specificity and PPV compared with universal thresholds.^41,42^ Further research is required to explore the efficacy of such approaches and understand the effectiveness of integration into clinical practice.

Our study has several strengths. The enrollment of consecutive patients using clinician suspicion of acute coronary syndrome eliminates selection bias. This ensured that our analysis included a wide range of patients, representative of the changing demographics observed in clinical practice, including more than a thousand patients aged >90 years, a group largely excluded from cardiovascular studies. A further strength is the adjudication of myocardial infarction according to the Fourth Universal Definition of Myocardial Infarction, in particular, given the increase in type 2 myocardial infarction and myocardial injury in older patients.

There are limitations that should be considered. Although our study reflected aging demographics, our local population is predominantly White, and findings may differ in a more ethnically diverse population. Our analysis was also based on cardiac troponin I measured using the Abbott ARCHITECT_STAT_ high-sensitivity assay. The 99th centile is assay dependent. Cardiac troponin I and T are not biologically equivalent neither is their relationship to age or cardiovascular risk.^3^ Our findings must therefore be interpreted with caution when considering other cardiac troponin assays. However, reduced performance with advancing age has now been observed in both high-sensitivity cardiac troponin I and T assays.^16^ We also recognize the challenge of diagnostic adjudication using routine health care data, in particular, in the older population where diagnostic procedures such as coronary angiography are performed less frequently.

In conclusion, age has a significant effect on the diagnostic performance of cardiac troponin at the guideline-recommended 99th centile for myocardial infarction, with reduced performance in older patients. The use of age-adjusted 99th centile thresholds or a higher universal rule-in threshold did not achieve parity between middle-aged and older patients. Individualized diagnostic approaches and serial testing to determine absolute change in troponin concentration rather than adjustment of binary thresholds are needed to avoid disadvantaging older patients.

## Article Information

### Acknowledgments

The authors thank the High-STEACS Investigators for their contributions to the conception or design of the work, or the acquisition, analysis, or interpretation of data for the work.

### Sources of Funding

This work was supported by the British Heart Foundation (grant number SP/12/10/29922) with support from a Research Excellence Award (grant number RE/18/5/34216). Drs Lowry, Doudesis, Wereski, and Bularga and are supported by Clinical Research Training Fellowships (grant numbers MR/W000598/1, MR/N013166/1, MR/V007017/1, and MR/V007254/1, respectively) from the Medical Research Council. Dr Lee is supported by a Clinical Research Training Fellowship (grant number FS/18/25/33454) from the British Heart Foundation. Dr Kimenai is supported by a grant from Health Data Research UK, which receives its funding from HDR UK Ltd (grant number HDR-5012) funded by the UK Medical Research Council, Engineering and Physical Sciences Research Council, Economic and Social Research Council, Department of Health and Social Care (England), Chief Scientist Office of the Scottish Government Health and Social Care Directorates, Health and Social Care Research and Development Division (Welsh Government), Public Health Agency (Northern Ireland), British Heart Foundation and the Wellcome Trust. Dr Newby is supported by the British Heart Foundation (grant number CH/09/002, RG/16/10/32375, RE/18/5/34216) and is the recipient of a Wellcome Trust Senior Investigator Award (grant number WT103782AIA). Dr Mills is supported by a Chair Award (grant number CH/F/21/90010), a Programme Grant (grant number RG/20/10/34966), and a Research Excellence Award (grant number RE/18/5/34216) from the British Heart Foundation. Dr Chapman receives support from a Starter Grant for Clinical Lecturers by the Academy of Medical Sciences (grant number SGL021/1075). Abbott Laboratories provided cardiac troponin assay reagents, calibrators, and controls without charge. The funders played no role in the design, conduct, data collection, analysis or reporting of the trial.

### Disclosures

Dr Mills reports research grants awarded to the University of Edinburgh from Abbott Diagnostics and Siemens Healthineers outside the submitted work, and honoraria from Abbott Diagnostics, Siemens Healthineers, Roche Diagnostics, and LumiraDx. The other authors report no conflicts.

### Supplemental Material

Tables S1–S6

Figures S1–S3

Methods

## Appendix

### Data Availability Statement

The High-STEACS trial makes use of several routine electronic health care data sources that are linked, de-identified, and held in our national safe haven, which is accessible by approved individuals who have undertaken the necessary governance training. Summary data can be made available on request to the corresponding author.

## Supplementary Material


