# Supplementary material for: Influence of age on the diagnosis of myocardial infarction
Source: Circulation. Author manuscript; Available in PMC 2022 Oct 19. (PMC9555758; doi:10.1161/CIRCULATIONAHA.122.059994)
Supplement: Influence of Age on the Diagnosis of Myocardial Infarction Supplementary Material [file EMS153392-supplement-Influence_of_Age_on_the_Diagnosis_of_Myocardial_Infarction_Supplementary_Material.pdf]

SUPPLEMENTAL MATERIAL

Influence of age on the diagnosis of myocardial infarction

Matthew TH Lowry MD[1], Dimitrios Doudesis MSc[1,2], Ryan Wereski MD[1], Dorien M Kimenai PhD[1], Christopher Tuck BSc[1], Amy V Ferry PhD[1], Anda Bularga MD,[1] Caelan Taggart MD[1], Kuan K Lee MD[1], Andrew R Chapman MD PhD[1], Anoop S.V. Shah MD PhD,[3,4] David E. Newby MD PhD[1], Nicholas L Mills MD PhD[1,2], Atul Anand MD PhD[1]
on behalf of the High-STEACS Investigators

[1] BHF Centre for Cardiovascular Science, University of Edinburgh, Edinburgh, UK
[2] Usher Institute, University of Edinburgh, Edinburgh, UK
[3] Department of Non-communicable Disease, London School of Hygiene and Tropical Medicine, London, UK
[4] Department of Cardiology, Imperial College Healthcare NHS Trust, London, UK

Short Title: Age and cardiac troponin

Corresponding author:
Dr Atul Anand
BHF Centre for Cardiovascular Science
University of Edinburgh
Edinburgh EH16 4SA
United Kingdom
Telephone: +44 131 242 6515
Fax: +44 131 242 6379
Email: atul.anand@ed.ac.uk

SUPPLEMENTARY APPENDIX A

Figures and Tables

Supplementary Tables

Online Table 1: Baseline characteristics stratified by age.

Online Table 2: Diagnostic performance of presentation high sensitivity cardiac troponin I in patients with suspected acute coronary syndrome by age and threshold.

Online Table 3: Diagnostic performance of presentation high-sensitivity cardiac troponin at the sex-specific 99th centile in patients with suspected acute coronary syndrome and chest pain as the presenting symptom.

Online Table 4: Diagnostic performance of presentation high-sensitivity cardiac troponin at the sex-specific 99th centile for the diagnosis of type 1 myocardial infarction.

Online Table 5: Timing of serial samples by age group

Online Table 6: Diagnosis between discordant threshold groups by age

Supplementary Figures

Online Figure 1: Cardiac troponin >99th centile upper reference limit by age group in whole population

Online Figure 2: Diagnostic accuracy of the sex-specific 99th centile at presentation in patients with chest pain

Online Figure 3: Three panel forest plot displaying positive predictive value of presentation high-sensitivity cardiac troponin I with a sex-specific 99th centile diagnostic threshold stratified by age groups

Online Table 1: Baseline characteristics stratified by age

Age (years)	All (N = 46,435)	<40 (N = 5,454)	40-44 (N = 2,884)	45-49 (N = 4,041)	50-54 (N = 5,008)	55-59 (N = 4,856)	60-64 (N = 4,207)	65-69 (N = 4,216)	70-74 (N = 4,093)	75-79 (N = 4,230)	80-84 (N = 3,714)	85-89 (N = 2,376)	≥90 (N = 1,356)
**Patient demographics**													
Age	61 (±17)	31 (±6)	42 (±1)	47 (±1)	52 (±1)	57 (1±)	62 (±1)	67 (±1)	72 (±1)	77 (±1)	82 (±1)	87 (±1)	93 (±3)
Male	24,726 (53%)	3,323 (61%)	1,673 (58%)	2,207 (55%)	2,802 (56%)	2,690 (55%)	2,430 (58%)	2,311 (55%)	2,179 (53%)	2,045 (48%)	1,658 (45%)	946 (40%)	462 (34%)
Presenting symptom chest pain*	33,480 (83%)	4,357 (93%)	2,361 (92%)	3,271 (91%)	4,019 (90%)	3,767 (88%)	3,076 (83%)	2,926 (80%)	2,736 (77%)	2,733 (74%)	2,205 (70%)	1,335 (66%)	694 (60%)
**Time from chest pain onset to presentation**													
≤2hrs (Early)	7,767 (17%)	734 (13%)	447 (15%)	666 (16%)	862 (17%)	824 (17%)	770 (18%)	728 (17%)	716 (17%)	735 (17%)	656 (18%)	398 (17%)	231 (17%)
≥12hrs (Late)	14,406 (31%)	2,020 (37%)	1,031 (36%)	1,346 (33%)	1,689 (34%)	1,487 (31%)	1,320 (31%)	1,315 (31%)	1,169 (29%)	1,169 (28%)	952 (26%)	593 (25%)	315 (23%)
**Past medical history**													
Myocardial infarction	4,059 (9%)	61 (1%)	124 (4%)	239 (6%)	410 (8%)	458 (9%)	477 (11%)	429 (10%)	478 (12%)	530 (13%)	423 (11%)	277 (12%)	153 (11%)
Ischaemic heart disease	11,472 (25%)	92 (2%)	199 (7%)	449 (11%)	825 (16%)	1,092 (22%)	1,153 (27%)	1,306 (31%)	1,523 (37%)	1,753 (41%)	1,576 (42%)	977 (41%)	527 (39%)
Hypercholesterolaemia	18,603 (40%)	139 (3%)	335 (12%)	739 (18%)	1,393 (28%)	1,904 (39%)	2,034 (48%)	2,402 (57%)	2,643 (65%)	2,780 (66%)	2,305 (62%)	1,342 (56%)	587 (43%)
Cerebrovascular disease	2,767 (6%)	24 (<1%)	27 (1%)	58 (1%)	137 (3%)	180 (4%)	208 (5%)	293 (7%)	343 (8%)	470 (11%)	462 (12%)	353 (15%)	212 (16%)
Chronic kidney disease	9,828 (21%)	423 (8%)	205 (7%)	315 (8%)	511 (10%)	694 (14%)	740 (18%)	904 (21%)	1,193 (29%)	1,520 (36%)	1,547 (42%)	1,079 (45%)	697 (51%)

Diabetes mellitus	3,315 (7%)	19 (<1%)	50 (2%)	92 (2%)	203 (4%)	295 (6%)	368 (9%)	412 (10%)	498 (12%)	578 (14%)	456 (12%)	240 (10%)	104 (8%)
Heart failure	3,990 (9%)	42 (1%)	49 (2%)	105 (3%)	162 (3%)	225 (5%)	264 (6%)	373 (9%)	531 (13%)	690 (16%)	684 (18%)	506 (21%)	359 (26%)
Multimorbidity	14,590 (31%)	97 (2%)	214 (7%)	495 (12%)	903 (18%)	1,269 (26%)	1,374 (33%)	1,638 (39%)	2,005 (49%)	2,326 (55%)	2,136 (58%)	1,377 (58%)	756 (56%)
**Previous Revascularisation**													
Percutaneous coronary intervention	3,574 (8%)	44 (1%)	114 (4%)	231 (6%)	386 (8%)	467 (10%)	466 (11%)	462 (11%)	470 (11%)	430 (10%)	318 (9%)	143 (6%)	43 (3%)
Coronary artery bypass grafting	756 (2%)	<5 (<1%)	8 (<1%)	27 (1%)	31 (1%)	74 (2%)	83 (2%)	112 (3%)	129 (3%)	139 (3%)	107 (3%)	35 (1%)	10 (1%)
**Medications at presentation**													
Aspirin	12,650 (27%)	136 (2%)	232 (8%)	491 (12%)	894 (18%)	1,220 (25%)	1,336 (32%)	1,547 (37%)	1,738 (42%)	1,845 (44%)	1,652 (44%)	977 (41%)	582 (43%)
P2Y12 inhibitor	4,397 (9%)	45 (1%)	72 (2%)	164 (4%)	305 (6%)	390 (8%)	439 (10%)	492 (12%)	553 (14%)	649 (15%)	626 (17%)	434 (18%)	228 (17%)
Dual antiplatelet therapy†	1,559 (3%)	38 (1%)	47 (2%)	100 (2%)	158 (3%)	177 (4%)	201 (5%)	181 (4%)	176 (4%)	183 (4%)	155 (4%)	94 (4%)	49 (4%)
ACEi or ARB	14,981 (32%)	216 (4%)	394 (14%)	743 (18%)	1,254 (25%)	1,586 (33%)	1,643 (39%)	1,826 (43%)	1,975 (48%)	2,137 (51%)	1,762 (47%)	996 (42%)	449 (33%)
Beta-blocker	12,670 (27%)	411 (8%)	364 (13%)	636 (16%)	969 (19%)	1,236 (25%)	1,294 (31%)	1,505 (36%)	1,646 (40%)	1,762 (42%)	1,493 (40%)	887 (37%)	467 (34%)
Lipid lowering therapy	18,603 (40%)	139 (3%)	335 (12%)	739 (18%)	1,393 (28%)	1,904 (39%)	2,034 (48%)	2,402 (57%)	2,643 (65%)	2,780 (66%)	2,305 (62%)	1,342 (56%)	587 (43%)
Oral anticoagulation‡	3,088 (7%)	56 (1%)	41 (1%)	72 (2%)	103 (2%)	140 (3%)	205 (5%)	336 (8%)	462 (11%)	595 (14%)	575 (15%)	341 (14%)	162 (12%)
**Physiological parameters§**													
Heart rate, beats per minute	86 (±26)	85 (±26)	83 (±25)	84 (±23)	83 (±24)	84 (±26)	85 (±26)	88 (±31)	87 (±27)	86 (±26)	87 (±26)	86 (±26)	88 (±26)
Systolic blood pressure, mmHg	139 (±29)	134 (±22)	138 (±27)	139 (±28)	139 (±28)	142 (±28)	142 (±27)	140 (±28)	137 (±30)	138 (±29)	139 (±30)	141 (±29)	142 (±31)

GRACE score	142 (±37)	76 (±23)	85 (±19)	97 (±24)	104 (±22)	113 (±24)	124 (±25)	134 (±25)	147 (±30)	153 (±26)	163 (±29)	169 (±27)	175 (±27)
**Electrocardiogram§**													
Normal ECG	2,516 (37%)	84 (53%)	88 (60%)	123 (46%)	208 (47%)	247 (47%)	255 (46%)	280 (40%)	276 (35%)	286 (32%)	311 (31%)	204 (27%)	154 (29%)
Ischaemia on ECG	1,739 (26%)	22 (14%)	27 (18%)	83 (31%)	126 (29%)	144 (28%)	181 (32%)	195 (28%)	226 (29%)	204 (23%)	253 (25%)	161 (21%)	117 (22%)
ST-segment elevation	243 (4%)	19 (12%)	8 (5%)	16 (6%)	28 (6%)	21 (4%)	20 (4%)	17 (2%)	26 (3%)	26 (3%)	28 (3%)	20 (3%)	14 (3%)
ST-segment depression	1,185 (18%)	10 (6%)	11 (8%)	50 (19%)	74 (17%)	96 (18%)	116 (21%)	134 (19%)	167 (21%)	141 (16%)	188 (19%)	115 (15%)	83 (15%)
T-wave inversion	1,188 (18%)	25 (16%)	26 (18%)	54 (20%)	90 (21%)	112 (21%)	116 (21%)	130 (19%)	131 (17%)	157 (17%)	152 (15%)	120 (16%)	75 (14%)
**Haematology and clinical chemistry**													
Haemoglobin, g/L	136 (±21)	144 (±20)	142 (±20)	142 (±19)	141 (±19)	140 (±19)	138 (±20)	136 (±20)	133 (±22)	129 (±22)	126 (±21)	123 (±22)	120 (±22)
Estimated glomerular filtration rate, mL/min	88 (±24)	116 (±15)	106 (±15)	102 (±15)	98 (±15)	93 (±17)	88 (±18)	82 (±19)	76 (±20)	72 (±20)	67 (±20)	63 (±19)	58 (±19)
Presentation high sensitivity troponin I, ng/mL	3 [1-11]	1 [1-2]	1 [1-3]	1 [1-3]	2 [1-4]	2 [1-6]	3 [2-9]	4 [2-13]	6 [3-17]	7 [4-20]	10 [5-29]	13 [6-35]	17 [8-54]
Peak high sensitivity troponin I, ng/mL	4 [1-13]	1 [1-2]	1 [1-3]	2 [1-3]	2 [1-5]	3 [1-7]	4 [2-11]	5 [2-16]	7 [3-22]	8 [4-26]	11 [5-41]	15 [7-49]	20 [9-79]
Serial troponin measurement¶	22,162 (48%)	1,433 (26%)	1,168 (40%)	1,763 (44%)	2,409 (48%)	2,456 (51%)	2,151 (51%)	2,176 (52%)	2,187 (53%)	2,296 (54%)	2,101 (57%)	1,328 (56%)	694 (51%)
**Adjudicated Diagnosis**													
Myocardial Infarction	5,279 (11%)	76 (1%)	120 (4%)	246 (6%)	393 (8%)	483 (10%)	507 (12%)	589 (14%)	632 (15%)	670 (16%)	696 (19%)	481 (20%)	313 (23%)
Type 1 myocardial infarction	4,064 (9%)	53 (1%)	107 (4%)	218 (5%)	349 (7%)	425 (9%)	429 (10%)	478 (11%)	481 (12%)	498 (12%)	484 (13%)	323 (14%)	219 (16%)
Type 2 myocardial infarction	1,116 (2%)	23 (<1%)	13 (<1%)	23 (1%)	47 (1%)	59 (1%)	71 (2%)	106 (3%)	144 (4%)	168 (4%)	212 (6%)	156 (7%)	94 (7%)

Type 4b myocardial infarction	36 (<1%)	0 (0%)	0 (0%)	5 (<1%)	<5 (<1%)	<5 (<1%)	7 (<1%)	5 (<1%)	7 (<1%)	<5 (<1%)	0 (0%)	<5 (<1%)	0 (0%)
Acute myocardial injury	1,676 (4%)	54 (1%)	22 (1%)	35 (1%)	48 (1%)	72 (1%)	87 (2%)	140 (3%)	197 (5%)	250 (6%)	299 (8%)	262 (11%)	210 (15%)
Chronic myocardial injury	1,287 (3%)	49 (1%)	26 (1%)	27 (1%)	61 (1%)	61 (1%)	74 (2%)	101 (2%)	130 (3%)	190 (4%)	233 (6%)	187 (8%)	148 (11%)
No myocardial injury	38,256 (82%)	5,275 (97%)	2,716 (94%)	3,733 (92%)	4,500 (90%)	4,236 (87%)	3,539 (84%)	3,386 (80%)	3,134 (77%)	3,120 (74%)	2,486 (67%)	1,446 (61%)	685 (51%)

Presented as number (%), mean (±SD) or median [inter-quartile range]

Abbreviations: ACE = Angiotensin-converting enzyme; ARB = Angiotensin receptor blocker; GRACE = Global Registry of Acute Cardiac Events

*Chest pain as presenting symptom is reported for the 87% (40,475/46,435) of patients where primary symptom data was available

† Two medications from aspirin, clopidogrel, prasugrel and ticagrelor

‡ Includes warfarin or novel anticoagulants

§Electrocardiographic and physiological data reported for the 83% (6,762/8,179) patients with myocardial infarction or myocardial injury who had electrocardiographic data available.

¶Serial testing defined as two or more tests within 24 hours of presentation.

Online Table 2: Diagnostic performance of presentation high sensitivity cardiac troponin I in patients with suspected acute coronary syndrome by age and threshold (n=46,435).

Age group (years)	True positives	False positives	True negatives	False negatives	Sensitivity (95% CI)	Specificity (95% CI)	PPV (95% CI)	NPV (95% CI)	Rule-in (%)
Sex-specific 99th centile									
<40	63	101	5277	13	83.0 (74.3-90.7)	98.1 (97.8-98.5)	38.4 (31.3-46.1)	99.8 (99.6-99.9)	3.0
40-44	95	43	2721	25	79.1 (71.3-85.9)	98.4 (98.0-98.9)	68.8 (61.0-76.6)	99.1 (98.7-99.4)	4.8
45-49	188	59	3741	53	78.0 (72.2-82.9)	98.5 (98.0-98.8)	76.1 (70.4-81.1)	98.6 (98.2-98.9)	6.1
50-54	320	102	4510	76	80.8 (76.7-84.6)	97.8 (97.3-98.2)	75.8 (71.3-79.7)	98.3 (98.0-98.7)	8.4
55-59	398	122	4250	86	82.2 (78.9-85.7)	97.2 (96.7-97.7)	76.5 (72.9-80.0)	98.0 (97.6-98.4)	10.7
60-64	394	148	3559	106	78.9 (75.0-82.5)	96.0 (95.4-96.6)	72.7 (69.1-76.4)	97.1 (96.6-97.6)	12.9
65-69	473	218	3414	111	81.0 (77.8-84.2)	94.0 (93.2-94.7)	68.4 (64.9-72.0)	96.9 (96.3-97.4)	16.4
70-74	503	299	3169	122	80.5 (77.4-83.5)	91.4 (90.4-92.3)	62.8 (59.4-65.9)	96.3 (95.6-96.9)	19.6
75-79	525	406	3158	141	78.8 (75.6-81.9)	88.6 (87.6-89.7)	56.5 (53.1-59.7)	95.7 (95.1-96.4)	22.0
80-84	558	489	2529	138	80.2 (77.5-83.1)	83.8 (82.5-85.1)	53.3 (50.3-56.3)	94.8 (94.0-95.6)	28.2
85-89	401	417	1480	78	83.8 (80.5-86.9)	78.1 (76.1-79.9)	49.1 (45.8-52.5)	95.0 (93.9-96.1)	34.4
≥90	274	341	702	39	87.6 (84.0-91.2)	67.3 (64.5-70.1)	44.5 (40.6-48.5)	94.8 (93.2-96.3)	45.4
Age-adjusted thresholds									

<40	63	101	5277	13	83.0 (74.3-90.7)	98.1 (97.8-98.5)	38.4 (31.3-46.1)	99.8 (99.6-99.9)	3.0
40-44	95	43	2721	25	79.1 (71.3-85.9)	98.4 (98.0-98.9)	68.8 (61.0-76.6)	99.1 (98.7-99.4)	4.8
45-49	188	59	3741	53	78.0 (72.2-82.9)	98.5 (98.0-98.8)	76.1 (70.4-81.1)	98.6 (98.2-98.9)	6.1
50-54	320	102	4510	76	80.8 (76.7-84.6)	97.8 (97.3-98.2)	75.8 (71.3-79.7)	98.3 (98.0-98.7)	8.4
55-59	398	122	4250	86	82.2 (78.9-85.7)	97.2 (96.7-97.7)	76.5 (72.9-80.0)	98.0 (97.6-98.4)	10.7
60-64	367	138	3569	133	73.5 (69.6-77.4)	96.3 (95.7-96.9)	72.7 (69.0-76.5)	96.4 (95.8-97.0)	12.0
65-69	451	197	3435	133	77.2 (73.8-80.6)	94.6 (93.8-95.3)	69.6 (65.7-73.3)	96.3 (95.6-96.9)	15.4
70-74	342	160	3308	283	54.7 (51.1-58.7)	95.4 (94.7-96.1)	68.1 (64.3-71.9)	92.1 (91.2-93.1)	12.3
75-79	367	215	3349	299	55.1 (51.3-59.0)	94.0 (93.2-94.8)	63.2 (59.2-67.2)	91.8 (91.0-92.7)	13.8
80-84	388	252	2766	308	55.8 (52.0-59.5)	91.7 (90.6-92.6)	60.6 (57.1-64.3)	90.0 (88.9-91.0)	17.2
85-89	257	197	1700	222	53.7 (49.2-58.2)	89.7 (88.3-91.0)	56.7 (52.5-61.6)	88.5 (87.1-89.9)	19.1
≥90	191	163	880	122	61.0 (55.8-66.1)	84.4 (82.1-86.6)	53.9 (48.5-59.2)	87.8 (85.8-89.8)	26.1
Universal threshold >99th centile									
<40	43	62	5316	33	56.4 (45.6-68.1)	98.8 (98.6-99.1)	40.9 (31.6-50.6)	99.4 (99.2-99.6)	1.9
40-44	69	26	2738	51	57.5 (49.1-66.7)	99.1 (98.7-99.4)	72.7 (63.5-81.3)	98.2 (97.7-98.7)	3.3
45-49	146	37	3763	95	60.4 (54.1-66.4)	99.0 (98.7-99.3)	79.7 (73.8-85.1)	97.5 (97.0-98.0)	4.5

50-54	225	56	4556	171	56.8 (51.9-61.7)	98.8 (98.5-99.1)	80.0 (75.1-84.8)	96.4 (95.8-96.9)	5.6
55-59	285	56	4316	199	58.8 (54.3-63.1)	98.7 (98.4-99.0)	83.5 (79.5-87.4)	95.6 (95.0-96.2)	7.0
60-64	277	77	3630	223	55.5 (51.3-59.8)	97.9 (97.5-98.4)	78.3 (74.1-82.6)	94.2 (93.5-94.9)	8.4
65-69	314	110	3522	270	53.8 (49.8-58.0)	97.0 (96.4-97.5)	74.1 (69.9-78.3)	92.9 (92.1-93.7)	10.1
70-74	334	146	3322	291	53.4 (49.6-57.5)	95.8 (95.1-96.4)	69.6 (65.5-73.4)	91.9 (91.0-92.8)	11.7
75-79	332	172	3392	334	49.9 (46.1-53.7)	95.2 (94.5-95.9)	66.0 (61.6-70.4)	91.0 (90.2-91.9)	11.9
80-84	361	215	2803	335	51.9 (48.1-55.6)	92.9 (92.0-93.8)	62.7 (59.0-66.5)	89.3 (88.2-90.4)	15.5
85-89	222	167	1730	257	46.4 (41.9-51.0)	91.2 (90.1-92.5)	57.2 (52.5-62.0)	87.1 (85.6-88.6)	16.4
≥90	164	139	904	149	52.3 (46.9-57.5)	86.7 (84.5-88.8)	54.0 (48.4-59.8)	85.8 (83.8-87.9)	22.3

Presented as number or % (95% confidence intervals) as appropriate.
Sex-specific 99th centile = 34 ng/L men, 16 ng/L women.
Age-adjusted thresholds = age <60: >32ng/L men, >16ng/L women; age 60-69: > 42ng/L men, >17ng/L women; age ≥70: 86ng/L men, 39g/L women)
Uniform rule-in threshold >99th centile = >64 ng/L
Abbreviations: NPV = negative predictive value, PPV = positive predictive value, URL = upper reference limit

Online Table 3: Diagnostic performance of presentation high-sensitivity cardiac troponin at the recommended sex-specific 99th centile in patients with suspected acute coronary syndrome and chest pain as the presenting symptom (n=33,480).

Age group (years)	True positives	False positives	True negatives	False negatives	Sensitivity (95% CI)	Specificity (95% CI)	PPV (95% CI)	NPV (95% CI)	Rule-in (%)
<40	52	64	4229	12	81.3 (71.7-90.1)	98.5 (98.1-98.9)	44.6 (35.7-54.6)	99.7 (99.6-99.9)	2.7
40-44	74	25	2239	23	76.4 (67.8-84.0)	98.9 (98.4-99.3)	74.7 (66.0-83.3)	99.0 (98.5-99.4)	4.2
45-49	151	25	3047	48	75.9 (69.9-81.6)	99.2 (98.9-99.5)	85.7 (80.0-90.6)	98.4 (98.0-98.9)	5.4
50-54	277	64	3618	60	82.2 (77.9-86.2)	98.3 (97.8-98.7)	81.3 (77.3-85.5)	98.4 (97.9-98.7)	8.5
55-59	325	62	3303	77	80.8 (77.3-84.6)	98.2 (97.7-98.6)	84.1 (80.3-87.4)	97.7 (97.2-98.2)	10.3
60-64	314	63	2610	89	77.8 (74.0-81.7)	97.6 (97.1-98.2)	83.3 (79.1-87.0)	96.7 (96.0-97.3)	12.3
65-69	361	82	2396	87	80.5 (76.7-84.1)	96.7 (96.0-97.4)	81.5 (77.8-84.9)	96.5 (95.7-97.1)	15.1
70-74	392	116	2138	90	81.3 (77.6-84.6)	94.9 (94.0-95.8)	77.2 (73.5-80.9)	95.9 (95.2-96.8)	18.6
75-79	395	139	2078	121	76.5 (72.8-80.1)	93.7 (92.7-94.7)	74.0 (70.3-77.8)	94.5 (93.6-95.4)	19.5
80-84	413	159	1525	108	79.3 (76.0-82.8)	90.5 (89.2-91.9)	72.2 (68.4-75.7)	93.4 (92.2-94.6)	25.9
85-89	292	146	831	66	81.5 (77.7-85.5)	85.1 (82.9-87.3)	66.7 (62.3-71.3)	92.6 (91.1-94.4)	32.8

≥90	199	99	363	33	85.7 (81.0-90.1)	78.6 (75.1-82.1)	66.7 (61.7-72.3)	91.6 (89.0-94.1)	42.9
Overall	3245	1044	28377	814	79.9 (78.6-81.1)	96.5 (96.2-96.7)	75.6 (74.4-77.0)	97.2 (97.0-97.4)	12.8

Presented as number or % (95% confidence intervals) as appropriate.
Abbreviations: PPV = positive predictive value, URL = upper reference limit

Online Table 4: Diagnostic performance of presentation high-sensitivity cardiac troponin at the recommended sex-specific 99th centile for the diagnosis of type 1 myocardial infarction

Age group (years)	True positives	False positives	True negatives	False negatives	Sensitivity (95% CI)	Specificity (95% CI)	PPV (95% CI)	NPV (95% CI)	Rule-in (%)
Sex-specific 99th centile									
<40	45	119	5282	8	85.0 (74.5-94.0)	97.8 (97.4-98.1)	27.4 (20.8-34.1)	99.8 (99.8-99.9)	3.0
40-44	85	53	2724	22	79.3 (71.1-86.3)	98.1 (97.6-98.6)	61.6 (53.1-70.2)	99.2 (98.8-99.5)	4.8
45-49	171	76	3747	47	78.4 (72.5-83.7)	98.0 (97.6-98.4)	69.2 (63.2-74.8)	98.8 (98.4-99.1)	6.1
50-54	284	138	4521	65	81.4 (77.2-85.2)	97.0 (96.5-97.5)	67.3 (62.7-71.8)	98.6 (98.2-98.9)	8.4
55-59	351	169	4262	74	82.6 (78.9-86.1)	96.2 (95.6-96.7)	67.5 (63.6-71.5)	98.3 (97.9-98.7)	10.7
60-64	346	196	3582	83	80.7 (76.7-84.1)	94.8 (94.1-95.5)	63.9 (60.2-67.8)	97.7 (97.2-98.2)	12.9
65-69	391	300	3438	87	81.8 (78.3-85.1)	92.0 (91.1-92.8)	56.6 (52.9-60.3)	97.5 (97.0-98.0)	16.4
70-74	389	413	3199	92	80.9 (77.3-84.2)	88.6 (87.5-89.6)	48.5 (44.9-51.9)	97.2 (96.6-97.8)	19.6
75-79	394	537	3195	104	79.1 (75.3-82.8)	85.6 (84.5-86.7)	42.3 (39.2-45.7)	96.8 (96.2-97.4)	22.0
80-84	399	648	2582	85	82.5 (79.2-85.7)	80.0 (78.5-81.3)	38.2 (35.3-41.1)	96.8 (96.1-97.5)	28.2
85-89	268	550	1503	55	83.0 (78.7-86.9)	73.3 (71.2-75.1)	32.9 (29.6-35.9)	96.5 (95.5-97.3)	34.4

≥90	193	422	715	26	88.2 (83.8-92.3)	62.9 (60.3-65.7)	31.5 (27.8-35.3)	96.5 (95.1-97.8)	45.4
Overall	3316	3621	38750	748	81.6 (80.3-82.8)	91.5 (91.2-91.7)	47.8 (46.6-49.0)	98.1 (98.0-98.2)	14.9
Age-adjusted thresholds									
<40	45	119	5282	8	85.0 (74.5-94.0)	97.8 (97.4-98.1)	27.4 (20.8-34.1)	99.8 (99.8-99.9)	3.0
40-44	85	53	2724	22	79.3 (71.1-86.3)	98.1 (97.6-98.6)	61.6 (53.1-70.2)	99.2 (98.8-99.5)	4.8
45-49	171	76	3747	47	78.4 (72.5-83.7)	98.0 (97.6-98.4)	69.2 (63.2-74.8)	98.8 (98.4-99.1)	6.1
50-54	284	138	4521	65	81.4 (77.2-85.2)	97.0 (96.5-97.5)	67.3 (62.7-71.8)	98.6 (98.2-98.9)	8.4
55-59	351	169	4262	74	82.6 (78.9-86.1)	96.2 (95.6-96.7)	67.5 (63.6-71.5)	98.3 (97.9-98.7)	10.7
60-64	321	184	3594	108	74.9 (70.7-79.0)	95.1 (94.4-95.8)	63.6 (59.7-67.7)	97.1 (96.5-97.6)	12.0
65-69	373	275	3463	105	78.0 (74.2-81.6)	92.6 (91.8-93.4)	57.5 (53.9-61.5)	97.1 (96.5-97.6)	15.4
70-74	275	227	3385	206	57.2 (52.6-61.7)	93.7 (92.9-94.5)	54.8 (50.6-58.9)	94.3 (93.5-95.1)	12.3
75-79	284	298	3434	214	57.0 (52.4-61.4)	92.0 (91.1-92.9)	48.8 (44.8-52.8)	94.1 (93.4-94.9)	13.8
80-84	286	354	2876	198	59.1 (54.9-63.4)	89.0 (87.9-90.1)	44.7 (40.6-48.6)	93.6 (92.7-94.4)	17.2
85-89	181	273	1780	142	56.1 (50.8-61.4)	86.8 (85.3-88.2)	40.0 (35.7-44.8)	92.6 (91.4-93.7)	19.1

≥90	137	217	920	82	62.5 (56.4-68.5)	81.0 (78.7-83.2)	38.8 (33.8-43.8)	91.8 (90.1-93.4)	26.1
Overall	2793	2383	39988	1271	68.7 (67.3-70.1)	94.4 (94.2-94.6)	53.9 (52.6-55.4)	96.9 (96.8-97.1)	11.1
Universal threshold >99th centile									
<40	34	71	5330	19	64.1 (51.2-76.7)	98.7 (98.4-99.0)	32.4 (23.3-41.5)	99.6 (99.5-99.8)	1.9
40-44	62	33	2744	45	57.9 (48.5-66.7)	98.8 (98.4-99.2)	65.3 (55.6-75.0)	98.4 (97.9-98.9)	3.3
45-49	137	46	3777	81	62.8 (56.6-68.7)	98.8 (98.5-99.1)	74.8 (68.4-80.8)	97.9 (97.5-98.3)	4.5
50-54	211	70	4589	138	60.4 (55.0-65.7)	98.5 (98.1-98.8)	75.0 (69.9-79.9)	97.1 (96.6-97.5)	5.6
55-59	264	77	4354	161	62.0 (57.4-66.8)	98.3 (97.9-98.6)	77.4 (73.0-81.6)	96.4 (95.9-97.0)	7.0
60-64	248	106	3672	181	57.8 (53.3-62.6)	97.2 (96.6-97.7)	70.1 (65.4-74.7)	95.3 (94.6-95.9)	8.4
65-69	265	159	3579	213	55.4 (51.1-60.0)	95.7 (95.1-96.4)	62.5 (57.8-67.0)	94.4 (93.6-95.1)	10.1
70-74	270	210	3402	211	56.1 (51.7-60.8)	94.2 (93.4-94.9)	56.2 (51.7-60.4)	94.2 (93.4-94.9)	11.7
75-79	262	242	3490	236	52.6 (48.2-57.1)	93.5 (92.7-94.3)	52.1 (47.6-56.6)	93.7 (92.9-94.4)	11.9
80-84	275	301	2929	209	56.8 (52.4-61.1)	90.7 (89.7-91.7)	47.8 (43.8-51.7)	93.3 (92.4-94.2)	15.5
85-89	159	230	1823	164	49.2 (43.9-54.6)	88.8 (87.6-90.2)	41.0 (36.2-45.9)	91.7 (90.5-92.9)	16.4

≥90	121	182	955	98	55.2 (48.6-61.4)	84.0 (81.8-86.2)	40.0 (34.6-45.3)	90.7 (88.8-92.4)	22.3
Overall	2308	1727	40644	1756	56.8 (55.2-58.4)	95.9 (95.7-96.1)	57.2 (55.7-58.8)	95.9 (95.7-96.0)	8.7

Presented as number or % (95% confidence intervals) as appropriate.
Sex-specific 99th centile = 34 ng/L men, 16 ng/L women.
Age-adjusted thresholds = age <60: >32ng/L men, >16ng/L women; age 60-69: > 42ng/L men, >17ng/L women; age ≥70: 86ng/L men, 39g/L women)
Uniform rule-in threshold >99th centile = >64 ng/L
Abbreviations: NPV = negative predictive value, PPV = positive predictive value, URL = upper reference limit

Online Table 5: Timing of serial samples by age group (n=20,881)

**Age (years)**	**<50**<br>**N=3,962**	**50-75**<br>**N=10,826**	**≥ 75**<br>**N=6,093**	**Overall**<br>**N=20,881**
**Time from presentation troponin to repeat sample (hrs)**				
<3 hrs	778 (20%)	1,511 (14%)	554 (8.9)	2,833 (14%)
3-6 hrs	1,037 (26%)	2,208 (20%)	863 (14%)	4,108 (20%)
6-9 hrs	816 (21%)	2,585 (24%)	1,664 (27%)	5,065 (24%)
9-12 hrs	778 (20%)	2,674 (25%)	1,665 (27%)	5,117 (25%)
>12 hrs	553 (15%)	1,848 (17%)	1,357 (22%)	3,758 (18%)
Presented as number (%)				

Online Table 6: Diagnosis between discordant threshold groups. a) sex-specific 99th centile and age-adjusted; b)age-adjusted threshold and 64ng/L ; c) sex-specific 99th centile and 64ng/L

a) Patients with presentation troponin samples between the sex-specific 99th centile and age-adjusted threshold

**Age group**	**<50 (n=0)**	**50-75 (n=380)**	**>75 (n=1,381)**
Myocardial Infarction	n/a	210 (55%)	555 (40%)
Type 1	n/a	157 (41%)	366 (27%)
Type 2	n/a	53 (14%)	189 (14%)
Type 4 b/c	n/a	1 (0.3%)	3 (0.2%)
Acute myocardial injury	n/a	88 (23%)	454 (33%)
Chronic myocardial Injury	n/a	81 (21%)	369 (27%)

b) Patients with presentation troponin samples between the age-adjusted threshold and 64ng/L

**Age group**	**<50 (n=166)**	**50-75 (n=756)**	**>75 (n=387)**
Myocardial Infarction	88 (53%)	469 (62%)	167 (43%)
Type 1	68 (41%)	364 (48%)	100 (26%)
Type 2	20 (12%)	105 (14%)	67 (17%)
Type 4 b/c	0 (0%)	5 (0.7%)	1 (0.3%)
Acute myocardial injury	30 (18%)	139 (18%)	116 (30%)
Chronic myocardial Injury	48 (29%)	143 (19%)	103 (27%)

c) Patients with presentation troponin samples between the sex-specific 99th centile and 64ng/L

**Age group**	**<50 (n=166)**	**50-75 (n=1,097**	**>75 (n=1,639)**
Myocardial Infarction	88 (53%)	653 (60%)	679 (41%)
Type 1	68 (41%)	503 (46%)	437 (27%)
Type 2	20 (12%)	150 (14%)	242 (15%)
Type 4 b/c	0 (0%)	5 (0.5%)	4 (0.2%)
Acute myocardial injury	30 (18%)	221 (20%)	522 (32%)
Chronic myocardial Injury	48 (29%)	218 (20%)	434 (26%)

Online Figure 1: Cardiac troponin >99th centile upper reference limit by age group in whole population

Bar plot showing the proportion of patients with at least one cardiac troponin >99th centile upper reference limit by age group for the whole study population.

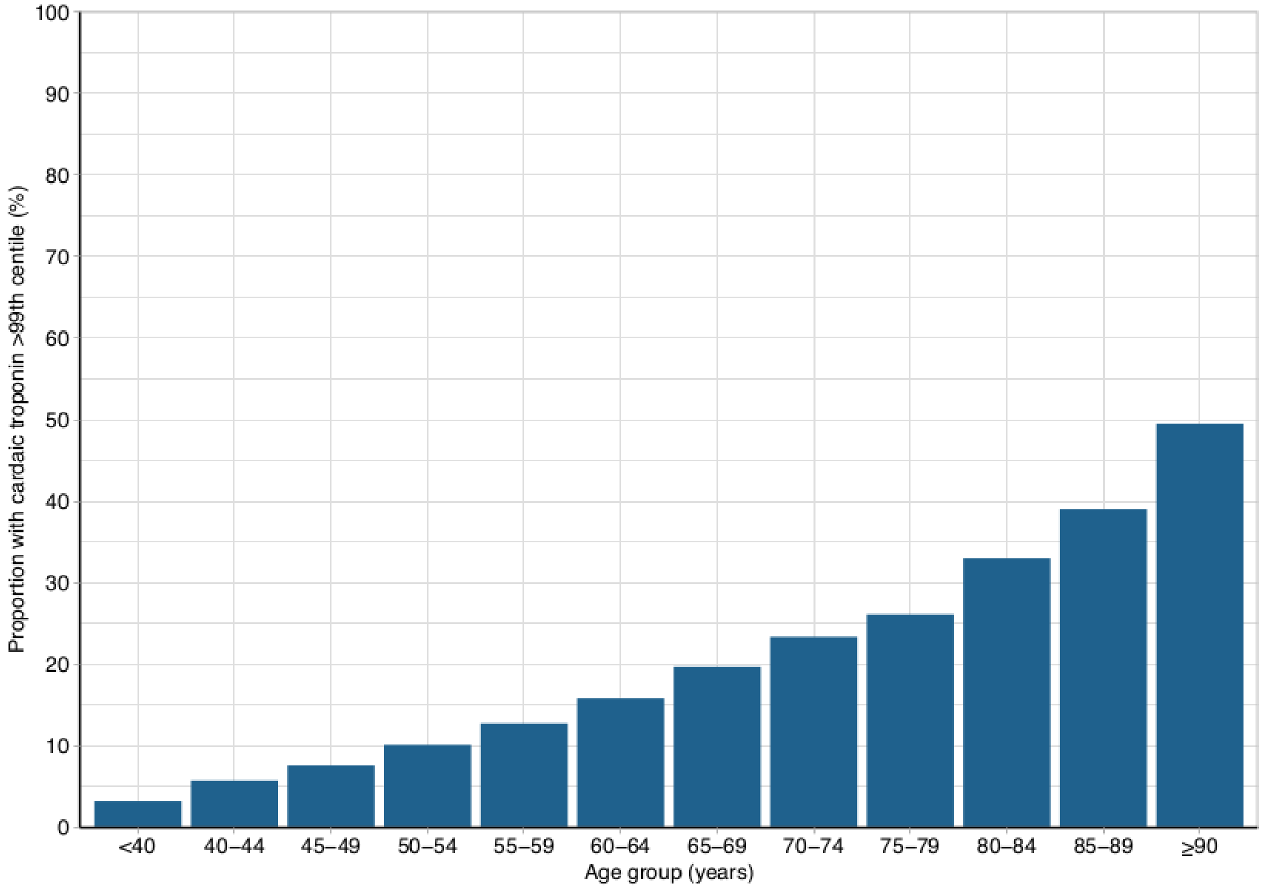

Online Figure 2: Diagnostic accuracy of the 99th centile at presentation in patients with chest pain

Line plot with error bars representing 95% confidence interval. The sensitivity, specificity and positive predictive value (PPV) of the 99th centile across age groups restricted to patients presenting with chest pain. (Online Table 3).

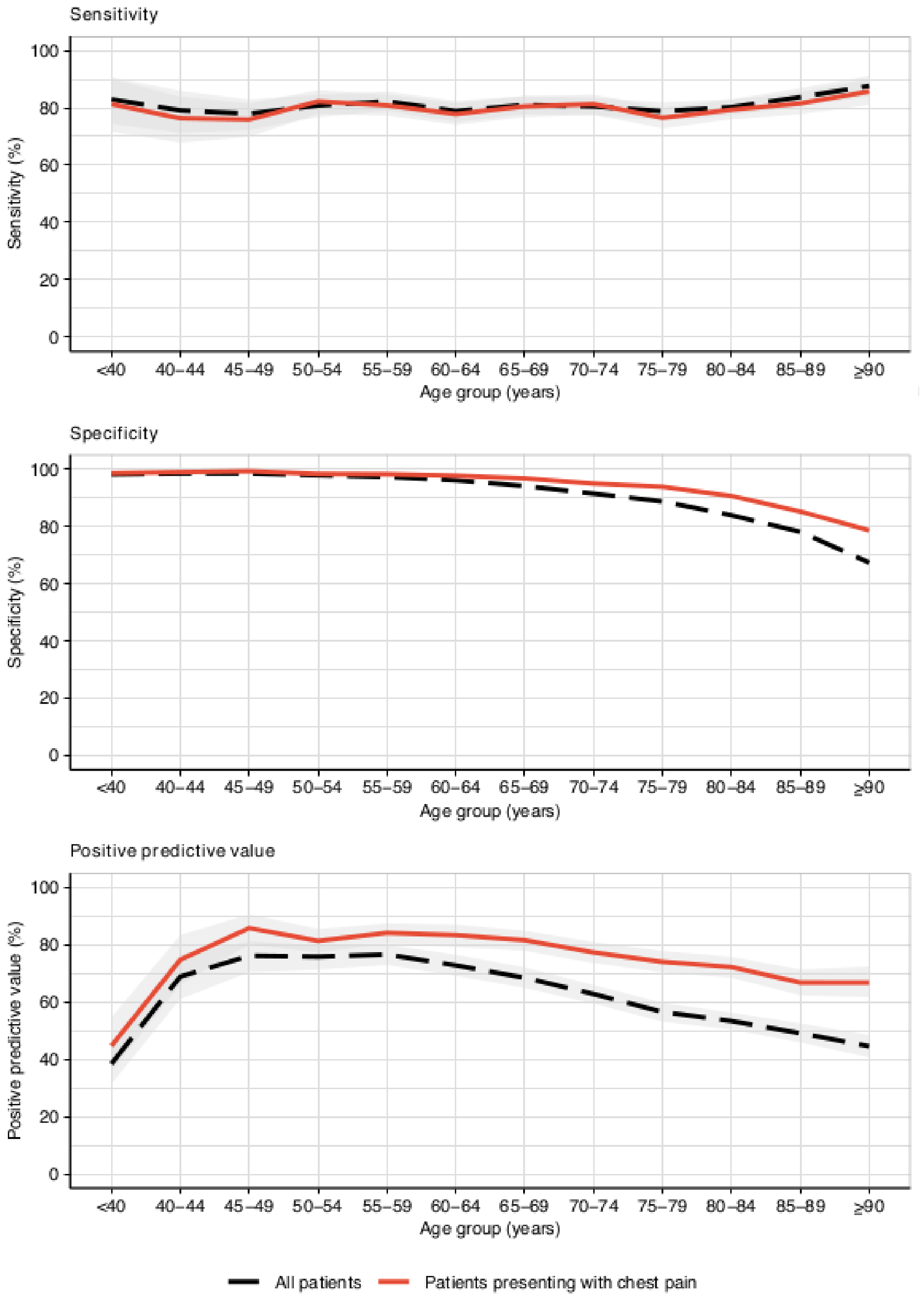

Online Figure 3: Three panel forest plot displaying positive predictive value of presentation high-sensitivity cardiac troponin I with a sex-specific 99th centile diagnostic threshold stratified by age groups

a) Age <50 years

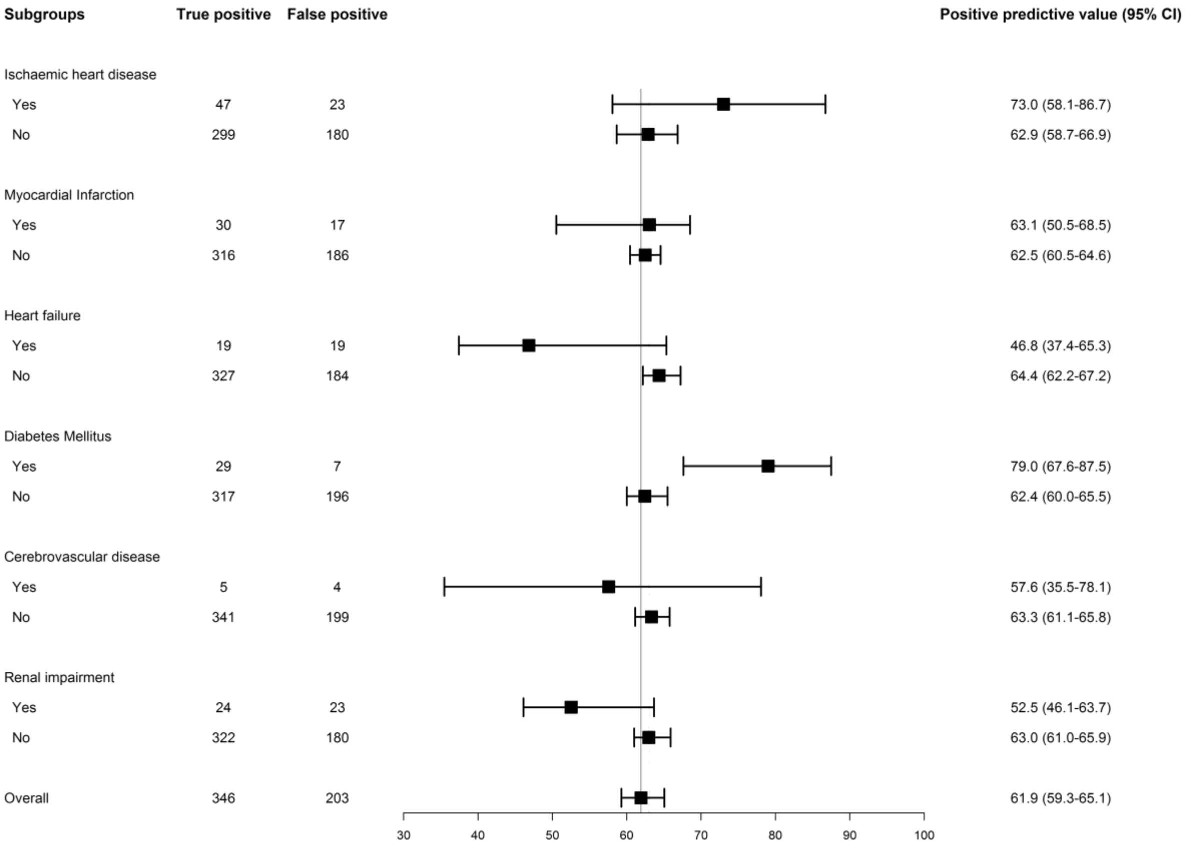

b) Age 50-74 years

Subgroups	True positive	False positive	Positive predictive value (95% CI)
Ischaemic heart disease			
Yes	577	310	65.4 (63.5-66.9)
No	1511	579	72.4 (70.7-74.3)
Myocardial Infarction			
Yes	265	137	66.4 (62.6-69.7)
No	1823	752	70.5 (69.5-71.4)
Heart failure			
Yes	281	204	56.8 (53.0-58.5)
No	1807	685	72.7 (71.4-74.3)
Diabetes Mellitus			
Yes	358	129	73.1 (69.9-77.2)
No	1730	760	69.7 (68.3-70.3)
Cerebrovascular disease			
Yes	124	94	55.7 (50.5-59.9)
No	1964	795	71.0 (70.6-71.9)
Renal impairment			
Yes	448	310	59.2 (55.7-62.6)
No	1640	579	73.7 (72.5-74.8)
Overall	2088	889	70.4 (68.6-71.7)

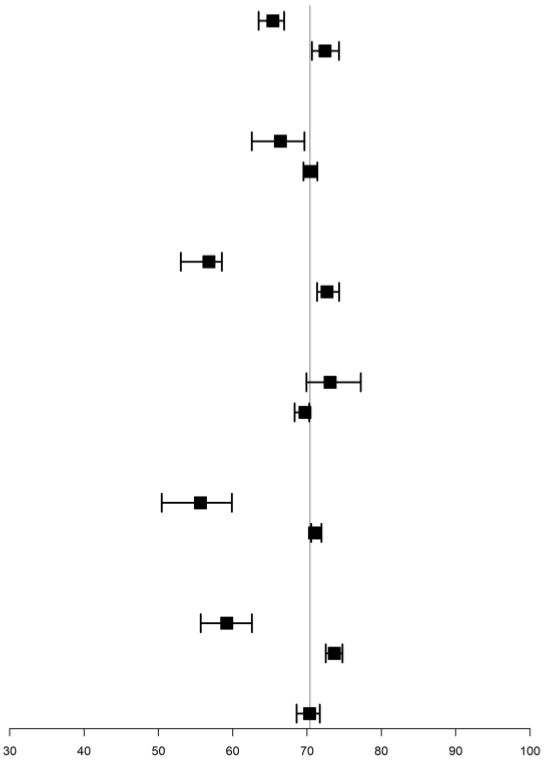

c) Age ≥75 years

Subgroups	True positive	False positive	Positive predictive value (95% CI)
Ischaemic heart disease			
Yes	786	622	55.3 (52.1-57.1)
No	972	1031	48.4 (47.3-50.3)
Myocardial Infarction			
Yes	282	204	57.5 (53.0-59.7)
No	1476	1449	50.9 (50.0-51.9)
Heart failure			
Yes	523	506	51.2 (48.5-53.9)
No	1235	1147	52.6 (51.2-54.1)
Diabetes Mellitus			
Yes	276	210	56.8 (53.5-59.4)
No	1482	1443	50.4 (49.6-51.9)
Cerebrovascular disease			
Yes	239	235	50.0 (46.1-53.2)
No	1519	1418	51.5 (49.9-53.5)
Renal impairment			
Yes	692	810	47.2 (45.7-48.5)
No	1066	843	55.7 (54.7-56.4)
Overall	1758	1653	51.4 (50.2-52.6)

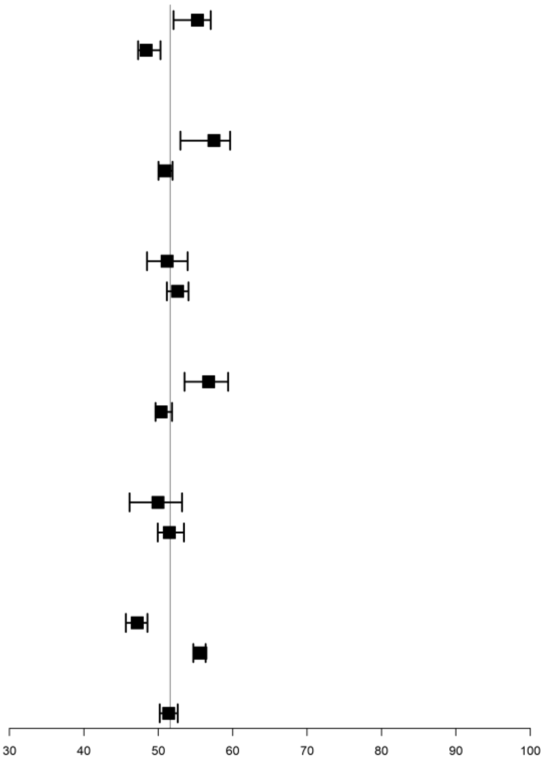

SUPPLEMENTARY APPENDIX B

Methodology

Adjudication according to the Fourth Universal Definition of Myocardial Infarction

All patients with high-sensitivity cardiac troponin I (hs-cTnI) concentrations above the sex-specific 99th centile were classified according to the Third Universal Definition of Myocardial Infarction in use at the time of the trial. In this pre-specified secondary analysis, we updated this classification in accordance with the Fourth Universal Definition of Myocardial Infarction. The final diagnosis was adjudicated according to a pre-specified list (cardiac diagnoses: acute aortic dissection, acute heart failure, cardiomyopathy, chronic heart failure, hypertensive heart disease, myopericarditis, non-ST segment elevation myocardial infarction, ST-segment elevation myocardial infarction, recent myocardial infarction, tachyarrhythmia, Takotsubo cardiomyopathy or valvular heart disease; non-cardiac diagnoses: acute kidney injury, chronic kidney disease, chronic obstructive pulmonary disease, gastrointestinal bleed, pulmonary embolism, sepsis, or other). Two physicians independently reviewed all clinical information, blinded to study phase, with discordant diagnoses resolved by a third reviewer. Clinical information included the dates and times of presentation and final discharge, the initial emergency department assessment and final discharge letter as documented in the electronic care record, with summaries of all investigations undertaken during the index presentation including the electrocardiogram. The adjudication panel had access to raw clinical information including haemoglobin, creatinine and high-sensitivity cardiac troponin I concentrations, and the reports from invasive coronary angiography. Type 1 myocardial infarction was defined as myocardial necrosis (any hs-cTnI concentration above the 99th centile with a rise and/or fall in hs-cTnI concentration where serial testing was performed) in the context of a presentation with suspected acute coronary syndrome with symptoms or signs of myocardial ischemia on

the electrocardiogram. Patients with symptoms or signs of myocardial ischemia and evidence of increased oxygen demand or decreased supply (for example, tachyarrhythmia, hypotension, or anaemia) secondary to an alternative pathology and myocardial necrosis were defined as type 2 myocardial infarction. The classification of type 2 myocardial infarction also includes patients with coronary vasospasm, embolism or spontaneous dissection without evidence of atherothrombosis related to coronary artery disease. Type 4a myocardial infarction was defined in patients with symptoms or signs of myocardial ischemia following percutaneous coronary intervention where hs-cTnI concentrations were 5-fold greater than the 99th centile, or increased further if elevated prior to the procedure. Type 4b myocardial infarction was defined where myocardial ischemia and myocardial necrosis were associated with stent thrombosis documented at angiography. Myocardial injury was defined if hs-cTnI concentrations were above the 99th centile in the absence of any clinical features of myocardial ischemia. Myocardial ischaemia was defined as All non-ischemic myocardial injury was classified as acute, unless a change of <20% was observed on serial testing or the final adjudicated diagnosis was chronic heart failure or chronic renal failure, where the classification was chronic myocardial injury.

Transparency and openness

The High-Sensitivity Troponin in the Evaluation of Patients with Suspected Acute Coronary Syndrome (High-STEACS) trial makes use of multiple routine electronic health care data sources that are linked, deidentified, and held in our national safe haven, which is accessible by approved individuals who have undertaken the necessary governance training. Summary data and the analysis code can be made available upon request from the corresponding author.

High-STEACS Trial Investigators

Chief Investigator: Prof Nicholas L Mills.

Trial managers: Dr Fiona E Strachan and Mr Christopher Tuck.

Trial research team: Dr Anoop SV Shah, Dr Atul Anand, Ms Amy V Ferry, Dr Kuan Ken Lee, Dr Andrew R Chapman, Dr Dimitrios Doudesis, Dr Anda Bularga, Dr Ryan Wereski, Dr Caelan Taggart, Dr Matthew TH Lowry, Mr Filip Mendusic, Dr Dorien M Kimenai, Mr Dennis Sandeman, Dr Philip D Adamson, Dr Catherine L Stables, Dr Catalina A Vallejo, Dr Athanasios Tsanas, Ms Lucy Marshall, Ms Stacey D Stewart, Ms Kelly Williams, Ms Grace Souter, Ms Jen Blades, Mr Andrew Sorbie, Dr Takeshi Fujisawa, Ms Mischa Hautvast, Ms Jean McPherson and Ms Lynn McKinlay.

Grant applicants: Prof Nicholas L Mills (Principal Applicant), Prof David E Newby, Prof Keith AA Fox, Prof Colin Berry, Dr Simon Walker, and Dr Christopher J Weir.

Trial steering committee: Prof Ian Ford (chair, independent), Prof Nicholas L Mills, Prof David E Newby, Prof Alasdair Gray, Prof Keith AA Fox, Prof Colin Berry, Dr Simon Walker, Prof Paul O Collinson, Prof Fred S Apple, Mr Alan Reid, Dr Anne Cruikshank, Dr Iain Findlay, Dr Shannon Amoils (independent), Dr David A McAllister, Dr Donogh Maguire, Ms Jennifer Stevens (independent), Prof John Norrie (independent), and Prof Christopher Weir.

Adjudication panel: Dr Anoop SV Shah, Dr Atul Anand, Dr Andrew R Chapman, Dr Kuan Ken Lee, Dr Jack PM Andrews, Dr Philip D Adamson, Dr Alastair Moss, Dr Mohamed S Anwar, Dr John Hung, Prof Nicholas L Mills.

Biochemistry sub-group committee: Dr Simon Walker, Dr Jonathan Malo, Mr Alan Reid, Dr Anne Cruikshank, Prof Paul O Collinson.

Data monitoring committee: Prof Colin M Fischbacher, Dr Bernard L Croal, Prof Stephen J Leslie.

Edinburgh Clinical Trials Unit: Ms Catriona Keerie, Mr Richard A Parker, Mr Allan Walker, Mr Ronnie Harkess, Mr Christopher Tuck, Mr Tony Wackett, Prof Christopher Weir.

NHS Greater Glasgow & Clyde Safe Haven: Dr Roma Armstrong, Ms Marion Flood, Ms Laura Stirling, Ms Claire MacDonald, Mr Imran Sadat, Mr Frank Finlay.

NHS Lothian Research Governance, eHealth and Safe Haven: Dr Heather Charles, Ms Pamela Linksted, Mr Stephen Young, Mr Bill Alexander, Mr Chris Duncan.
